# Cholesterol sulfate alleviates ulcerative colitis by promoting cholesterol biosynthesis in colonic epithelial cells

**DOI:** 10.1038/s41467-022-32158-7

**Published:** 2022-07-30

**Authors:** Dongke Xu, Ruijun Ma, Yi Ju, Xiaowei Song, Baolin Niu, Wenting Hong, Rong Wang, Qin Yang, Zhi Zhao, Yuchen Zhang, Yufan Zheng, Qianming Bai, Mingfang Lv, Ning Sun, Xiaobo Li

**Affiliations:** 1 Department of Physiology and Pathophysiology, School of Basic Medical Sciences, Fudan University, Shanghai, China; 2 Center for Gastrointestinal Endoscopy, Shanxi Provincial People’s Hospital, Taiyuan, China; 3 Department of Chemistry, Fudan University, Shanghai, China; 4 Department of Pathology, Shanxi Provincial People’s Hospital, Taiyuan, China; 5 Department of Medical Record and Statistics, Shanxi Provincial People’s Hospital, Taiyuan, China; 6 Department of Pathology, Fudan University Shanghai Cancer Centre, Shanghai, China; 7 Department of Immunology, School of Basic Medical Sciences, Fudan University, Shanghai, China; 8 Wuxi School of Medicine, Jiangnan University, Jiangsu, China

**Keywords:** Ulcerative colitis, Cell biology

## Abstract

Cholesterol sulfate, produced by hydroxysteroid sulfotransferase 2B1 (SULT2B1), is highly abundant in the intestine. Herein, we study the functional role and underlying intestinal epithelial repair mechanisms of cholesterol sulfate in ulcerative colitis. The levels of cholesterol and cholesterol sulfate, as well as the expression of *Sult2b1* and genes involved in cholesterol biosynthesis, are significantly higher in inflamed tissues from patients with ulcerative colitis than in intestinal mucosa from healthy controls. Cholesterol sulfate in the gut and circulation is mainly catalyzed by intestinal epithelial SULT2B1. Specific deletion of the *Sult2b1* gene in the intestinal epithelial cells aggravates dextran sulfate sodium-induced colitis; however, dietary supplementation with cholesterol sulfate ameliorates this effect in acute and chronic ulcerative colitis in mice. Cholesterol sulfate promotes cholesterol biosynthesis by binding to Niemann-Pick type C2 protein and activating sterol regulatory element binding protein 2 in colonic epithelial cells, thereby alleviates ulcerative colitis. In conclusion, cholesterol sulfate contributes to the healing of the mucosal barrier and exhibits therapeutic efficacy against ulcerative colitis in mice.

## Abstract (web summary)

New treatment strategies are required for ulcerative colitis. Here the authors show in mouse models that cholesterol sulfate, an endogenous active cholesterol derivative, contributes to the healing of the mucosal barrier by promoting cholesterol biosynthesis in colonic epithelial cells and exhibits therapeutic efficacy against ulcerative colitis.

## Introduction

Ulcerative colitis (UC), one of the most common forms of inflammatory bowel disease (IBD), is a chronic relapsing-remitting inflammatory disease that affects the colon, resulting in disability and requires lifelong treatment^1^. The incidence of UC has increased worldwide over the last few decades. Patients with UC exhibit mucosal inflammation, beginning in the rectum and extending to the proximal segments of the colon. Defects in the colonic epithelial barrier are strongly implicated in the pathogenesis of UC. Therefore, the aim for treatment of UC is to induce and maintain remission, including resolution of clinical symptoms and endoscopic mucosal healing. Currently, the main treatment options for UC are 5-aminosalicylic acid drugs, steroids, immunosuppressants, and biological drugs^2^. Despite the variety of available therapeutic options, unmet needs persist in terms of efficacy and safety^3^, and therefore, new treatment strategies with different mechanisms of action are required.

As an essential building block for membrane biosynthesis, cholesterol is irreplaceable for rapidly proliferating cells to sustain their growth^4,5^. In addition, cholesterol drives the mechanistic target of rapamycin complex 1 (mTORC1) activation and growth signaling^6,7^. Repair and reconstruction of the colonic epithelium during mucosal healing relies on sufficient supply of cholesterol. However, due to the lack of Niemann–Pick C1-like 1 (NPC1L1) protein in colonic epithelial cells^8^, cholesterol may not be absorbed from the intestinal lumen but from the plasma lipoprotein or de novo synthesis^9^. Increasing levels of cellular cholesterol stimulate crypt organoid growth and drive endogenous cholesterol synthesis by activating sterol regulatory element binding protein 2 (SREBP2), which promotes intestinal stem cell proliferation^10^. As the intestinal epithelium renews rapidly, SREBP2 is constitutively activated to meet the requirements for intestinal crypt proliferation^11^. However, the regulatory mechanism of cholesterol metabolism to meet these requirements during mucosal healing in UC is currently unknown. Controlling cholesterol metabolism to promote colonic epithelial cell proliferation may be a new treatment strategy for UC.

Cholesterol sulfate (CS) is a sulfated derivative of cholesterol catalyzed by hydroxysteroid sulfotransferase 2B1 (SULT2B1)^12,13^. Our previous study, in addition to others, found that SULT2B1 promoted liver regeneration in a partial hepatectomy (PH) model, increased hepatic oval cell proliferation in a chemical-induced liver injury model, and accelerated the growth of multiple cells^14–17^. CS is critical in several biological processes, including epidermal differentiation, sperm capacitation, platelet adhesion, blood clotting, fibrinolysis, gluconeogenesis, T cell receptor signaling, and cell invasion^18–22^. High abundance of CS is present in the intestinal contents, including feces, in rodents and humans. Moreover, 2–20% of the total cholesterol in feces is CS^23,24^. It is unclear whether CS is just a metabolic waste product of cholesterol eliminated via feces or whether CS plays important physiological and pathophysiological roles in the maintenance of intestinal homeostasis.

Cholesterol is delivered to the cells by low-density lipoprotein (LDL) receptor-mediated endocytosis of LDL particles. The LDL particles are transported to the lysosomes, where cholesteryl esters are hydrolyzed and the liberated free cholesterol is further utilized, in addition to assisting in feedback regulation of cholesterol biosynthesis and LDL uptake. Niemann–Pick type C 1 (NPC1) and C 2 (NPC2) proteins are required for cholesterol trafficking from endosome or lysosome to other intracellular compartments^25^. NPC2, a critical lysosomal cholesterol solubilizing and transport factor, extracts cholesterol from intralysosomal membranes and transfers it to NPC1. Ramirez et al. reported that Npc1 or Npc2 deletion sequestered sterols, mainly in the liver. Meanwhile, although not as colossal as in the liver, free cholesterol and sterol biosynthesis in the intestine were also increased in *Npc1*^−/−^ or *Npc2*^−/−^ mice^26^. The crystal structure of NPC2 bound to CS has been previously reported^27^. NPC2 binds to CS with a greater affinity than cholesterol; CS competes with cholesterol for binding to NPC2 but not NPC1^28–31^. Given that CS is highly abundant in the intestinal epithelium and mucosal healing of UC requires sufficient cholesterol, we proposed that CS, which is capable of binding to NPC2, may participate in intracellular cholesterol trafficking and metabolism in intestinal epithelial cells (IECs).

In this study, the levels of SUL2B1 and CS were detected in inflamed tissues from patients with UC. Next, the functional role and underlying intestinal epithelial repair mechanisms of CS in colitis were elucidated using dextran sulfate sodium (DSS)-challenged intestinal epithelial cell-specific *Sult2b1* deletion mice (*Sult2b1*^∆IEC^) and littermate control *Sult2b1*^f/f^ mice that were fed a diet supplemented with CS or cholesterol. We regularly monitored the clinical signs of colitis and analyzed whether a CS-supplemented diet could alleviate DSS-induced acute or chronic colitis or spontaneous colitis in interleukin (IL)−10-deficient mice. Further, we studied the ability of CS to bind to NPC2, activate SREBP2, and promote cholesterol biosynthesis and colonic epithelial cell proliferation.

## Results

### SULT2B1 and CS levels are increased in UC mucosa

**Fig. 1:**
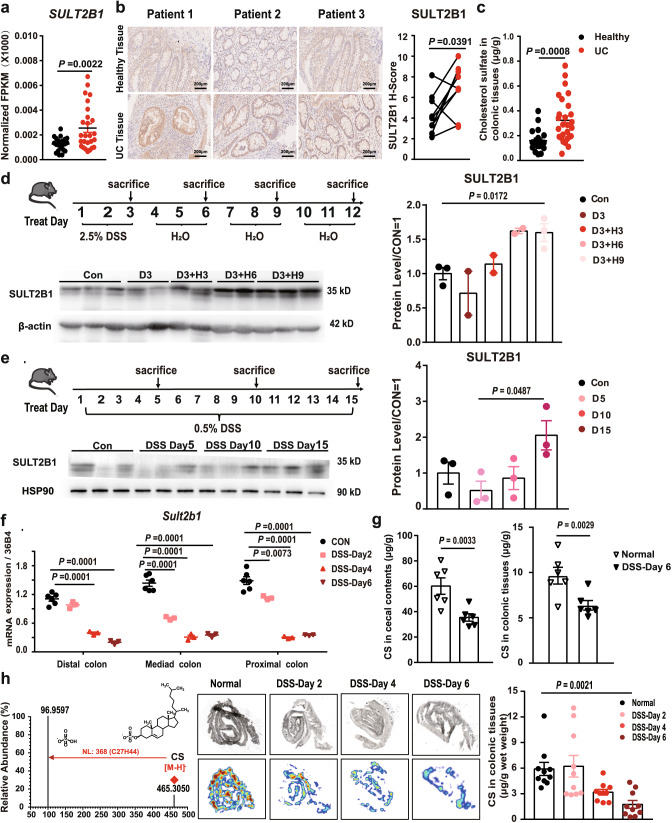
SULT2B1 and cholesterol sulfate (CS) levels are increased in ulcerative colitis (UC) mucosa. **a** Normalized FPKM (fragments per kilobase of transcript per million mapped reads) of *SULT2B1* in inflamed rectum mucosa of 26 patients with UC and 22 healthy controls based on RNA-seq data in GSE 111889. (Two-tailed Student’s *t* test). **b** SULT2B1 protein expression in the uninflamed and inflamed colonic mucosa from the same patients with UC using immunohistochemistry (*n* = 9, two-tailed, Wilcoxon matched-pairs signed rank test). **c** Quantification of CS in colonic tissues extracted by colonoscopy from healthy controls (*n* = 19) and patients with UC (*n* = 27) using LC–MS. CS concentration was calculated from the linear regression equation plotted from the standard curve and normalized with wet weight of colonic tissues. (Two-tailed Student’s *t* test). **d** Western blotting analyses were performed for SULT2B1 protein in the colon of mice challenged with the following: 3-day 2.5% DSS; 3-day 2.5% DSS + 3-day H_2_O; 3-day 2.5% DSS + 6-day H_2_O; 3-day 2.5% DSS + 9-day H_2_O; (Con: control mice; D3: 3-day 2.5% DSS; D3 + H3: 3-day 2.5% DSS + 3-day H_2_O; D3 + H6: 3-day 2.5% DSS + 6-day H_2_O; D3 + H9: 3-day 2.5% DSS + 9-day H_2_O) (*n* = 3, 2, 2, 2, 3 mice/group, respectively, one-way ANOVA with Sidak’s multiple comparison test). **e** Western blotting analyses were performed for SULT2B1 protein in the colon of mice challenged with the following: 5-day 0.5% DSS; 10-day 0.5% DSS; 15-day 0.5% DSS; (D5: 5-day 0.5% DSS; D10: 10-day 0.5% DSS; D15: 15-day 0.5% DSS) (*n* = 3 mice/group, one-way ANOVA with Sidak’s multiple comparison test). **f** RT-qPCR analysis of *Sult2b1* mRNA in distal, media, and proximal colonic tissues with 2-, 4-, and 6-day 2.5% DSS challenge, respectively. (*n* = 6, 3, 3, 3 mice/group, respectively, one-way ANOVA with Sidak’s multiple comparison test). **g** Quantification of CS in cecal contents and colonic tissues from mice with 6-day 2.5% DSS challenge and control group using LC–MS. CS concentration was calculated from the linear regression equation plotted from the standard curve and normalized with wet weight of cecal contents or colonic tissues. (*n* = 6 mice/group, two-tailed Student’s *t* test). **h** Quantification of CS in colonic tissues from mice with 2-, 4-, and 6-day 2.5% DSS challenge using DESI-MSI. Cholesterol sulfate ion is at *m/z* 465.3044, the signal variation was normalized by the total ion current (TIC, range from *m/z* 400 to 500) pixel-by-pixel. The approximate lateral resolution is 200 μm (*n* = 5 mice/group, 2 fields/mouse, one-way ANOVA with Sidak’s multiple comparison test). Data from in vitro assays are reprehensive of at least three independent experiments. Data are shown as the mean ± SEM. Source data are provided as a Source Data file.

To explore the potential role of SULT2B1 in the pathogenesis of UC, we analyzed the published RNA-seq data from 26 patients with UC and 22 healthy controls (GSE 111889)^32^. *SULT2B1* was significantly upregulated in the inflamed rectum tissues of patients with UC (Fig. 1a), which was consistent with our immunohistochemical results obtained from clinically inflamed colon and non-involved regions of the same patient with UC (Fig. 1b, Supplementary Table 1). Furthermore, we collected endoscopic biopsies from 27 patients with UC and 19 healthy individuals. Using liquid chromatography–mass spectrometry (LC–MS) analysis, it was observed that the levels of CS were significantly higher in the inflamed colonic tissues from patients with UC than in colonic mucosa from healthy controls (Fig. 1c, Supplementary Table 2). To investigate the signal that induced *SULT2B1* expression in the inflamed colonic mucosa, human colon epithelial HT-29 cells were treated with tumor necrosis factor alpha (TNFα) or lipopolysaccharide (LPS), which elucidated SULT2B1 to be upregulated at the mRNA level (Supplementary Fig. 1a–b).

Next, we monitored the dynamic changes in SULT2B1 expression in the colonic tissues of mice with DSS-induced colitis. In the mice recovered from 3-day 2.5% DSS challenge or mice with 0.5% DSS-induced colitis, the protein level of SULT2B1 in the colonic tissues was increased compared to that in controls (Fig. 1d–e). However, in the acute and severe mouse colitis model induced using 2.5% DSS treatment for six days, SULT2B1 expression was found to be decreased in colonic epithelia compared to that in controls (Fig. 1f, Supplementary Fig. 1c). Similarly, we performed LC–MS and desorption electrospray ionization–mass spectroscopy imaging (DESI–MSI) to measure the abundance of CS in the intestine before and after the 2.5% DSS challenge. The amount of CS significantly decreased in the inflamed colonic tissues and cecal contents in 2.5% DSS-fed mice compared to that in control mice (Fig. 1g–h, Supplementary Fig. 1d). The decrease in SULT2B1 and CS might be due to the notably high amount of colonic epithelial cell death and detachment observed in this acute and severe mouse colitis model.

Therefore, the increase in SULT2B1 and CS in the UC mucosa may play important roles in the pathogenesis of UC.

### Intestinal epithelial cell-derived or exogenous CS protects against 2.5% DSS-induced colitis

**Fig. 2:**
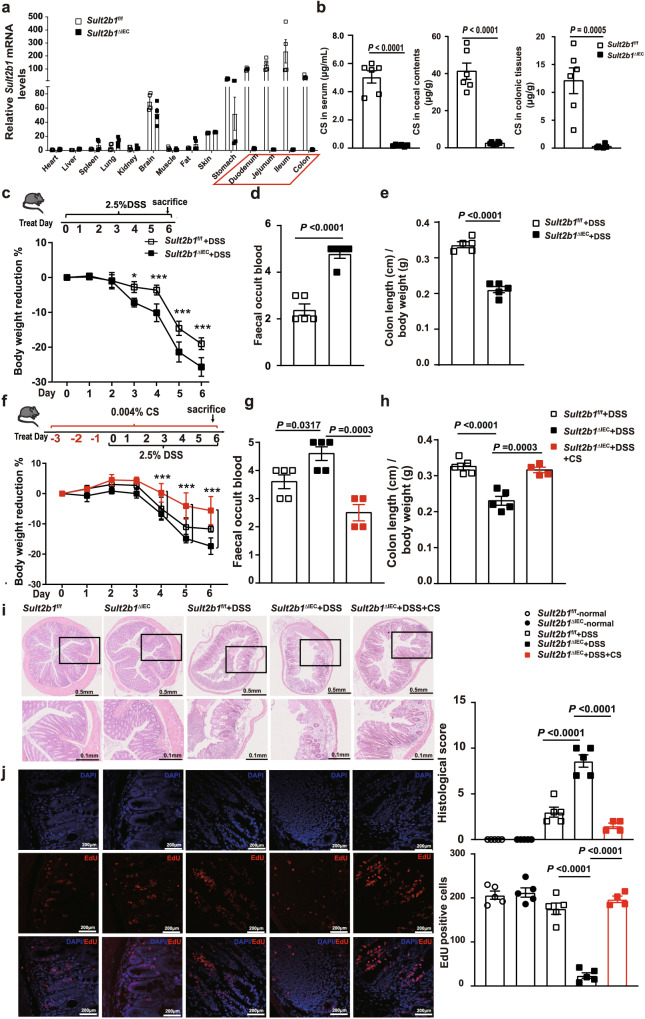
Intestinal epithelial cell-derived or exogenous CS is protective against 2.5% DSS-induced colitis. **a** RT-qPCR analysis of *Sult2b1* mRNA in different organs from *Sult2b1*^f/f^ and *Sult2b1*^∆IEC^ mice (*n* = 4 mice/group). **b** Quantification of CS in serum, cecal contents, and colonic tissues from *Sult2b1*^f/f^ and *Sult2b1*^∆IEC^ mice using LC–MS (*n* = 6 mice/group, two-tailed Student’s *t* test). **c** Schematic diagram of treatment and body weight reduction rate of *Sult2b1*^f/f^ and *Sult2b1*^∆IEC^ mice during DSS challenge (*n* = 5 mice/group), two-way ANOVA with Tukey’s multiple comparisons test, day 3 *P = 0.0139; day 4 ***P < 0.0001; day 5 ***P < 0.0001; day 6 ***P < 0.0001. **d**, **e** Faecal occult blood (FOB) of *Sult2b1*^f/f^ and *Sult2b1*^∆IEC^ mice on day 3, and colon length/body weight of these mice on day 6 of DSS challenge (*n* = 5 mice/group, two-tailed Student’s *t* test). **f** Schematic diagram of treatment and body weight reduction rate of *Sult2b1*^f/f^, *Sult2b1*^∆IEC^, and CS-fed *Sult2b1*^∆IEC^ mice during DSS challenge (*n* = 5, 5, 4 mice/group, respectively, two-way ANOVA with Tukey’s multiple comparisons test, day 4 (KO vs. KO + CS) ***P = 0.0003; day 5 (KO vs. KO + CS) ***P < 0.0001; day 6 (KO vs. KO + CS) ***P < 0.0001. **g**, **h** FOB and colon length/body weight of *Sult2b1*^f/f^, *Sult2b1*^∆IEC^, and CS-fed *Sult2b1*^∆IEC^ mice during DSS challenge (*n* = 5, 5, 4 mice/group, respectively, one-way ANOVA with Sidak’s multiple comparison test). **i**, **j**: Hematoxylin and eosin- and EdU-stained colonic sections excised from the same parts (2 cm away from the anus) of normal *Sult2b1*^f/f^, *Sult2b1*^∆IEC^, 6-day DSS-challenged *Sult2b1*^f/f^, *Sult2b1*^∆IEC^, and CS-fed *Sult2b1*^∆IEC^ mice (*n* = 5, 5, 5, 5, 4 mice/group, respectively, one-way ANOVA with Sidak’s multiple comparison test). Data are shown as the mean ± SEM. Source data are provided as a Source Data file.

To further study the physiological role of CS, we generated intestinal epithelial cell-specific *Sult2b1* deletion mice (*Sult2b1*^∆IEC^ mice) (Supplementary Fig. 1e). *Sult2b1* mRNA expression was diminished in jejunal, ileal, and colonic tissues; however, this was not observed in other tissues of the mice, thereby confirming the deletion specificity (Fig. 2a, Supplementary Fig. 1f). The growth and development of *Sult2b1*^∆IEC^ mice were normal, and the body weight and colon length were comparable between *Sult2b1*^∆IEC^ and *Sult2b1*^f/f^ mice of the same sex and age (Supplementary Fig. 1g). LC–MS analysis revealed that the CS levels in serum, colonic tissues, and cecal contents in *Sult2b1*^∆IEC^ mice were only 4.2%, 6.0%, and 2.3% of the levels in *Sult2b1*^f/f^ mice (control), respectively (Fig. 2b). These results show that the intestinal epithelial cells are the major source of CS in the intestinal tract and blood.

Following 2.5% DSS challenge, *Sult2b1*^∆IEC^ mice exhibited greater body weight loss (Fig. 2c) and faecal occult blood (FOB) than the control *Sult2b1*^f/f^ littermates (Fig. 2d). In addition, the colon length of *Sult2b1*^∆IEC^ mice were shortened (Fig. 2e). However, supplementation with 0.004% CS in the diet ameliorated body weight loss, FOB, and shortening of colon length in *Sult2b1*^∆IEC^ mice challenged with 2.5% DSS (Fig. 2f–h). Histological analysis showed that *Sult2b1*^∆IEC^ mice developed severe colitis, characterized by altered intestinal architecture, including villous damage, crypt loss, and extensive immune cell infiltration in the colon, which was prevented by supplementation with 0.004% CS (Fig. 2i). *Sult2b1*^∆IEC^ mice had fewer EdU-positive gut epithelial cells than the control mice; however, supplementation with 0.004% CS in the diet increased the number of EdU-positive colonic epithelial cells in *Sult2b1*^∆IEC^ mice (Fig. 2j).

In summary, intestinal epithelial cell-derived or exogenous CS protects against colitis in mice, likely by promoting epithelial cell proliferation and barrier repair.

### CS is required for cholesterol biosynthesis in colonic epithelial cells

**Fig. 3:**
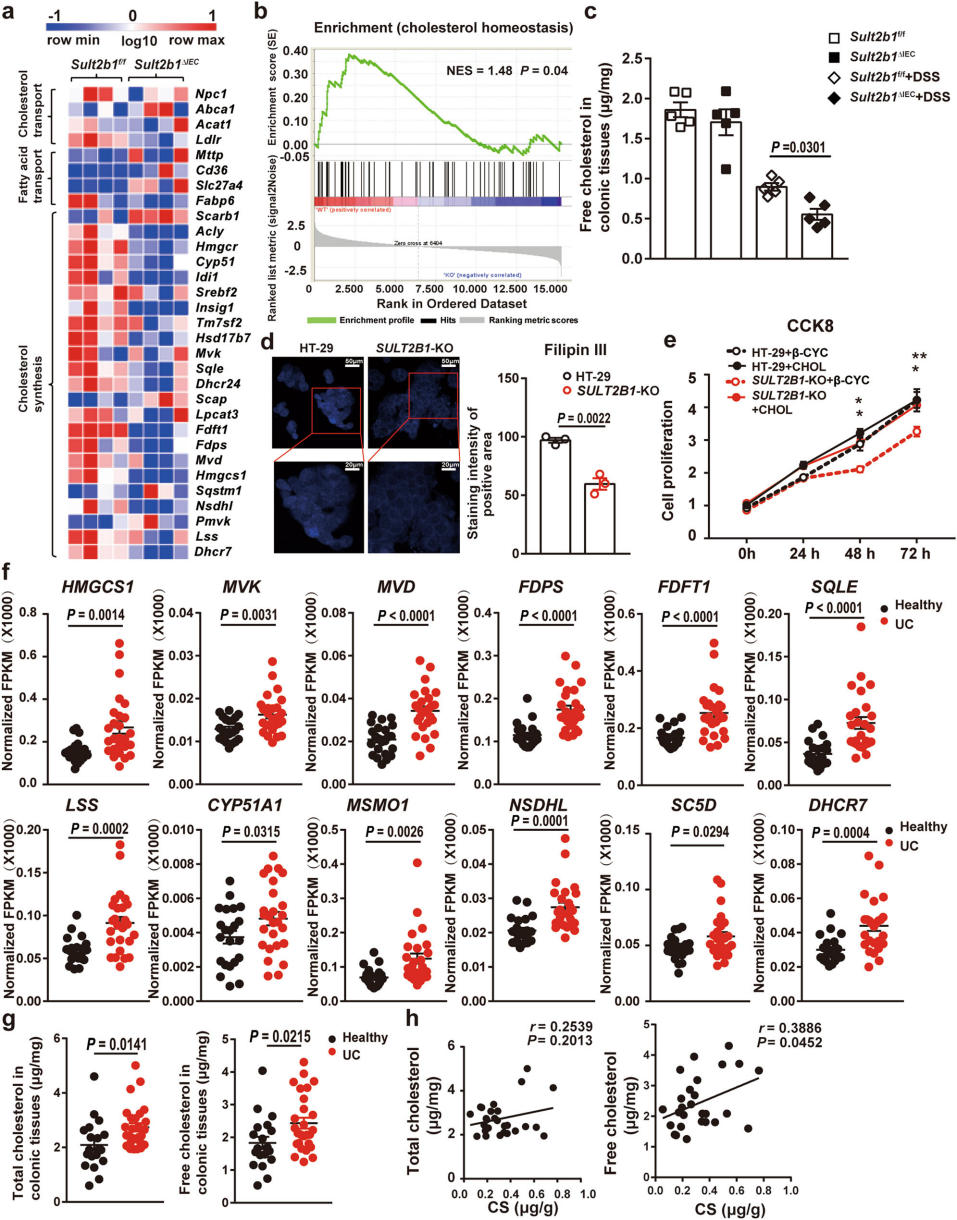
CS is required for cholesterol biosynthesis in colonic epithelial cells. **a** The heatmap shows the relative levels of cholesterol metabolism-related genes in inflamed colonic tissues from 6-day 2.5% DSS-treated *Sult2b1*^f/f^ and *Sult2b1*^∆IEC^ mice. (Fold change ≥ 2, *P* < 0.05) (*n* = 4 mice/group). **b** Gene Set Enrichment Analysis shows the enrichment of the cholesterol synthesis gene signature in the downregulated genes (*Sult2b1*^∆IEC^ vs. *Sult2b1*^f/f^ mice). **c** Quantification of free cholesterol concentration in colonic tissues from *Sult2b1*^f/f^ and *Sult2b1*^∆IEC^ mice in the presence or absence of 6-day 2.5% DSS challenge using the Amplex® Red Cholesterol Assay Kit (*n* = 5 mice/group, one-way ANOVA with Sidak’s multiple comparison test). **d** Filipin III staining in HT-29 and *SULT2B1*-KO cells. The boxed areas are shown in the lower panels. (*n* = 3 fields/group, two-tailed Student’s *t* test). **e** CCK-8 assay shows the proliferation of HT-29 and *SULT2B1*-KO HT-29 cells in the presence or absence of cholesterol (50 μM) dissolved in β-cyclodextrin (CHOL: cholesterol; β-CYC: β-cyclodextrin). Two-way ANOVA with Tukey’s multiple comparisons test, 48 h (HT-29+β-CYC vs. *SULT2B1*-KO + β-CYC, *P = 0.0209; *SULT2B1*-KO + β-CYC vs. *SULT2B1*-KO + CHOL, *P = 0.0127), 72 h (HT-29+β-CYC vs. *SULT2B1*-KO + β-CYC, **P = 0.0012; *SULT2B1*-KO + β-CYC vs. *SULT2B1*-KO + CHOL, *P = 0.0122). **f** Normalized FPKM of the cholesterol biosynthesis genes in inflamed rectum tissues of 26 patients with UC and 22 healthy controls based on RNA-seq data in GSE 111889. (Two-tailed Student’s t test). **g** Quantification of total and free cholesterol in colonic tissues extracted by colonoscopy from healthy controls (*n* = 19) and patients with UC (*n* = 27) using the Amplex® Red Cholesterol Assay Kit. (Two-tailed Student’s *t*-test). **h** Pearson’s correlation analysis was performed between CS and total cholesterol levels, or CS and free cholesterol levels in colonic tissues from 27 patients with UC. (Two-tailed *P* value). Data from in vitro assays are reprehensive of at least three independent experiments. Data are shown as the mean ± SEM. Source data are provided as a Source Data file.

To determine the molecular basis of SULT2B1 and CS in regulating colonic colitis, we performed RNA-seq using inflamed colon samples from *Sult2b1*^f/f^ and *Sult2b1*^∆IEC^ mice challenged with 2.5% DSS. The transcriptomic profile of colonic tissues in DSS-treated *Sult2b1*^∆IEC^ mice was significantly different from that in DSS-treated *Sult2b1*^f/f^ mice (Supplementary Fig. 2a, b). When compared with *Sult2b1*^f/f^ mice, 1507 genes were upregulated and 1106 were downregulated by at least 2-fold in *Sult2b1*^∆IEC^ mice (Supplementary Fig. 2c). Notably, the expression of genes important for cholesterol biosynthesis, including *Hmgcr*, *Hmgcs1*, *Cyp51*, *Fdft1, Fdps*, and *Mvd*, was downregulated in *Sult2b1*^∆IEC^ mice (Fig. 3a). Gene set enrichment analysis (GSEA) showed that cholesterol homeostasis (Normalized Enrichment Score (NES) = 1.48, *P* = 0.04) was reduced in *Sult2b1*^∆IEC^ mice (Fig. 3b). The colonic tissues of *Sult2b1*^f/f^ and *Sult2b1*^∆IEC^ mice had similar levels of cholesterol; however, the levels significantly decreased in *Sult2b1*^∆IEC^ mice following DSS challenge (Fig. 3c, Supplementary Fig. 2d).

To further study the role of CS in promoting cholesterol biosynthesis, we deleted the *SULT2B1* gene in human colon epithelial HT-29 cells (*SULT2B1*-KO) using the CRISPR-Cas9 technology. Successful *SULT2B1* gene deletion was confirmed using gene sequencing and western blotting (Supplementary Fig. 2e–f). RNA-seq was performed for wild-type HT-29 and *SULT2B1*-KO cells, and Gene Ontology enrichment analysis showed that the expression of genes involved in cholesterol biosynthesis was reduced in *SULT2B1*-KO cells (Supplementary Fig. 2g–h), which was consistent with the RNA-seq data obtained from the inflamed colonic tissues of *Sult2b1*^∆IEC^ and *Sult2b1*^f/f^ mice. Using Filipin III staining, we found that the level of free cholesterol in *SULT2B1*-KO cells was lower than that of wild-type cells, which was also consistent with the RNA-seq results (Fig. 3d). Cell proliferation was impaired by *SULT2B1* deletion, which was rescued by exogenous cholesterol in vitro (Fig. 3e). In addition, *SULT2B1*-KO cells were more sensitive to TNFα-induced apoptosis, while the addition of cholesterol prevented apoptosis (Supplementary Fig. 2i). Furthermore, addition of cholesterol dissolved in β-cyclodextrin promoted the growth of colonic organoids (Supplementary Fig. 3a, c).

We also analyzed the published RNA-seq data from 26 patients with UC and 22 healthy controls (GSE 111889)^28^. Most of the cholesterol biosynthesis genes, including *HMGCS1*, *MVK*, *MVD*, *FDPS*, *FDFT1*, *SQLE*, *LSS*, *CYP51, MSMO1*, *NSDHL, SC5D*, and *DHCR7* were upregulated in the inflamed rectum tissues of patients with UC (Fig. 3f). Further, the inflamed rectum tissues from patients with UC had significantly higher levels of cholesterol than the rectal mucosa from healthy controls (Fig. 3g). As shown previously (Fig. 1a–c), the levels of SULT2B1 and CS were also elevated in inflamed tissues from patients with UC. CS levels were positively correlated with the free cholesterol levels in the colonic mucosa from patients with UC (*r* = 0.3886, *P* = 0.0452; Fig. 3h). By promoting cholesterol biosynthesis, the upregulation of SULT2B1 and CS might be an adaptive and protective response during UC.

Therefore, CS is required for cholesterol biosynthesis and cell proliferation in intestinal epithelial cells.

### Dietary supplementation of CS alleviates colitis in mice

Next, we explored whether dietary supply of cholesterol could alleviate colitis. We found that a high-cholesterol diet (HCD, 1.5% cholesterol in diet) did not alleviate DSS-induced colitis in both *Sult2b1*^f/f^ and *Sult2b1*^∆IEC^ mice. The clinical signs of colitis, including loss of body weight (Supplementary Fig. 4a), faecal occult blood (FOB), colon shortening (Supplementary Fig. 4b, c), and histological injuries (Supplementary Fig. 4d), were more severe in the HCD groups in both *Sult2b1*^f/f^ and *Sult2b1*^∆IEC^ mice than in the normal diet groups following 2.5% DSS challenge.

**Fig. 4:**
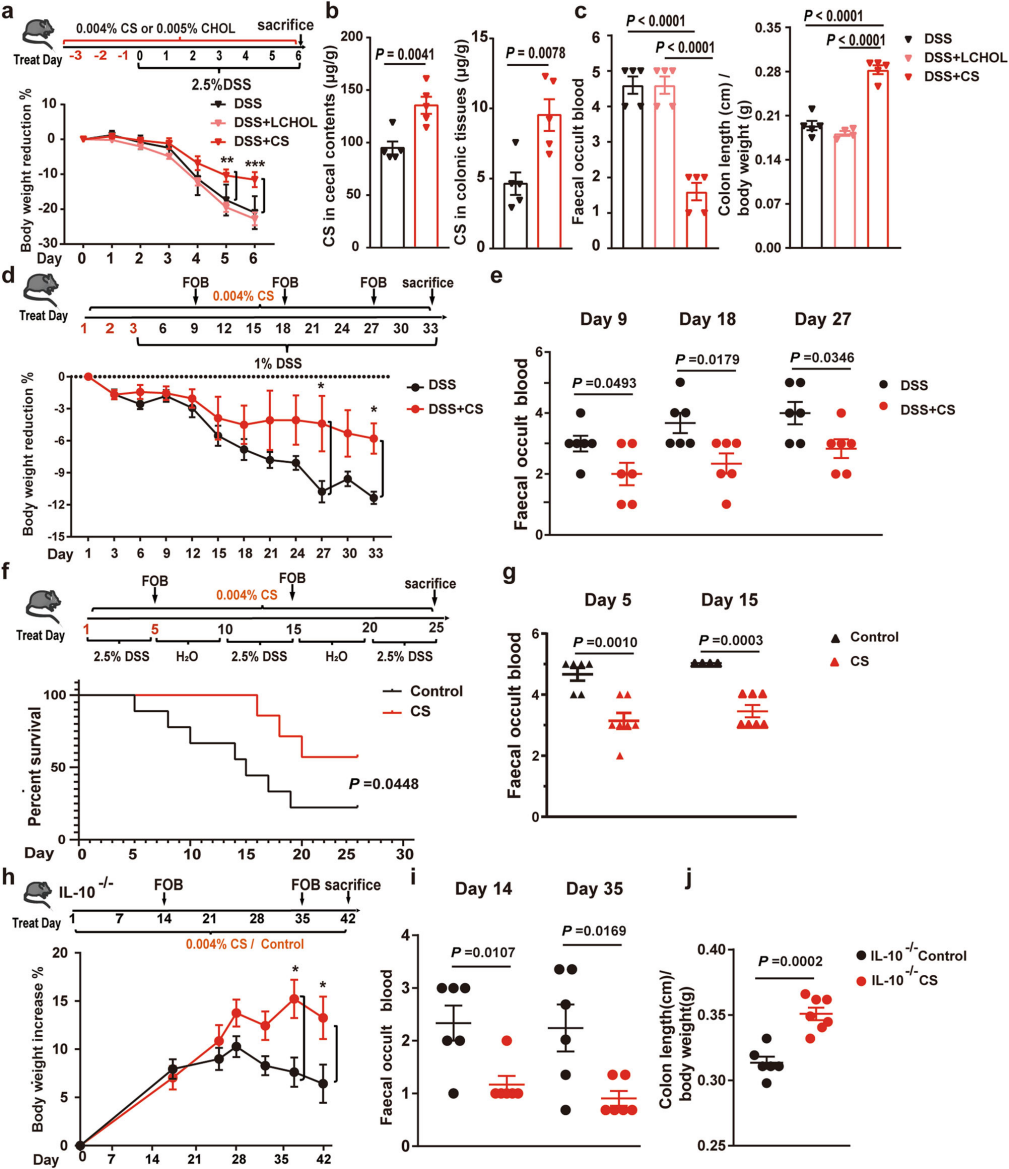
Dietary supplementation of CS, not cholesterol, alleviates colitis in mice. **a** Schematic diagram of the treatment and body weight loss rate of C57BL/6J, low dose cholesterol-fed (LCHOL-fed) C57BL/6J, and CS-fed C57BL/6J mice during the DSS challenge (*n* = 4 mice/group, LCHOL: low dose (0.005%) cholesterol). Two-way ANOVA with Tukey’s multiple comparisons test, day 5 (DSS vs. DSS + CS) **P = 0.0028; day 6 (DSS vs. DSS + CS) ***P < 0.0001. **b** Qualification of CS in cecal contents and colonic tissues from C57BL/6J and CS-fed C57BL/6J mice using LC–MS (*n* = 5 mice/group, two-tailed Student’s *t*-test). **c** FOB and colon length/body weight of C57BL/6J and CS-fed C57BL/6J mice (*n* = 5 mice/group, two-tailed Student’s *t* test). **d** Schematic diagram of the treatment and body weight loss rate of C57BL/6 mice challenged with 33-day 1% DSS and fed with or without 0.004% CS (*n* = 6 mice/group), two-way ANOVA with Tukey’s multiple comparisons test, day 27 (DSS vs. DSS + CS) *P = 0.0485; day 33 (DSS vs. DSS + CS) *P = 0.0319. **e** FOB of C57BL/6J mice challenged with 33-day 1% DSS and fed with or without 0.004% CS on the 9^th^, 18^th^, and 27^th^ day (*n* = 6 mice/group, two-tailed Student’s *t*-test). **f** Schematic diagram of the treatment and survival proportion of C57BL/6J mice challenged with 3-round 2.5% DSS and fed with or without 0.004% CS (Gehan-Breslow-Wilcoxon Test). **g** FOB of C57BL/6J mice challenged with 3-round 2.5% DSS and fed with or without 0.004% CS on the 5^th^ (*n* = 7, 6 mice/group, respectively) and 15^th^ (*n* = 7, 4 mice/group, respectively, two-tailed Student’s *t*-test). **h** Schematic diagram of the treatment and body weight increase rate of interleukin-10 deficient (IL-10^−/−^) mice fed with (*n* = 7) or without (*n* = 6) 0.004% CS. Two-way ANOVA with Tukey’s multiple comparisons test, day 37 (IL-10^−/−^Control vs. IL-10^−/−^CS) *P = 0.0338; day 42 (IL-10^−/−^Control vs. IL-10^−/−^CS) *P = 0.0437. **i** FOB of interleukin-10 deficient (IL-10^−/−^) mice fed with (*n* = 6) or without (*n* = 6) 0.004% CS on the 14^th^ day and 35^th^ day. (Two-tailed Student’s *t*-test). **j** Colon length/body weight of interleukin-10 deficient (IL-10^−/−^) mice fed with (*n* = 7) or without (*n* = 6) 0.004% CS. (Two-tailed Student’s *t*-test). Data from in vitro assays are reprehensive of at least three independent experiments. Data are shown as the mean ± SEM. Source data are provided as a Source Data file.

Further, C57BL/6J mice were fed a 0.004% CS or 0.005% cholesterol (having equal molar concentration with CS) diet and treated with 2.5% DSS, as indicated in the schematic diagram in Fig. 4a. As a result, CS content increased in cecal contents and in colonic tissues of 0.004% CS-fed mice (Fig. 4b). Meanwhile, the clinical signs of colitis showed a significant alleviation after CS treatment in 2.5% DSS-induced colitis mice model. However, supplementation with the same amount of cholesterol did not show protective effects (Fig. 4a, c, Supplementary Fig. 4e, f). No significant differences in food and water consumption were observed among the three groups, suggesting that the effects of CS were not due to altered nutrition or DSS uptake (Supplementary Fig. 4g, h). Moreover, studying the immune cells in the intestinal lamina propria elucidated that DSS induced immune cell recruitment and activation, while CS supplementation significantly reduced inflammation (Supplementary Fig. 4i, Supplementary Fig. 5a).

To obtain more information with respect to chronic colitis, we further studied the role of CS supplementation in chronic DSS-induced colitis and IL-10-knockout colitis mice model. In chronic colitis mice model induced by persistent 1% DSS feeding for 33 days, the clinical signs of colitis, including loss of body weight and FOB showed alleviation in CS treatment group compared with control group (Fig. 4d, e). In another chronic colitis mice model induced by intermittent 2.5% DSS feeding for three rounds separated by H_2_O treatment, the survival rate of the CS-fed mice was increased in comparison to that of control mice (Fig. 4f). Meanwhile, CS-treated mice also showed alleviated FOB compared with controls on the 5^th^ day and 15^th^ day (Fig. 4g).

The IL-10 deficient (IL-10^−/−^) mouse is another widely used chronic UC model. IL-10^−/−^ mice spontaneously develop UC starting at 2–3 months of age^33^. 8–10-week-old IL-10^−/−^ mice were fed with 0.004% CS or control diet for 42 days. As a result, the increase rate of body weight was enhanced by CS treatment (Fig. 4h). Meanwhile, the clinical signs of colitis, including FOB, colon shortening (Fig. 4i, j), and histological injuries (Supplementary Fig. 4j), were also alleviated by CS treatment. Meanwhile, the mRNA levels of IL-1β and IL-6 in colonic tissues were decreased by CS supplementation (Supplementary Fig. 4k). Furthermore, the CS-treated colonic organoids showed increased growth and budding compared with DMSO-treated controls (Supplementary Fig. 3b, c).

In summary, dietary supplementation of CS alleviates DSS-induced acute/chronic colitis and IL-10-deficient spontaneous colitis in mice.

### CS promotes cholesterol biosynthesis by binding to NPC2 and activating SREBP2 in colonic epithelial cells

**Fig. 5:**
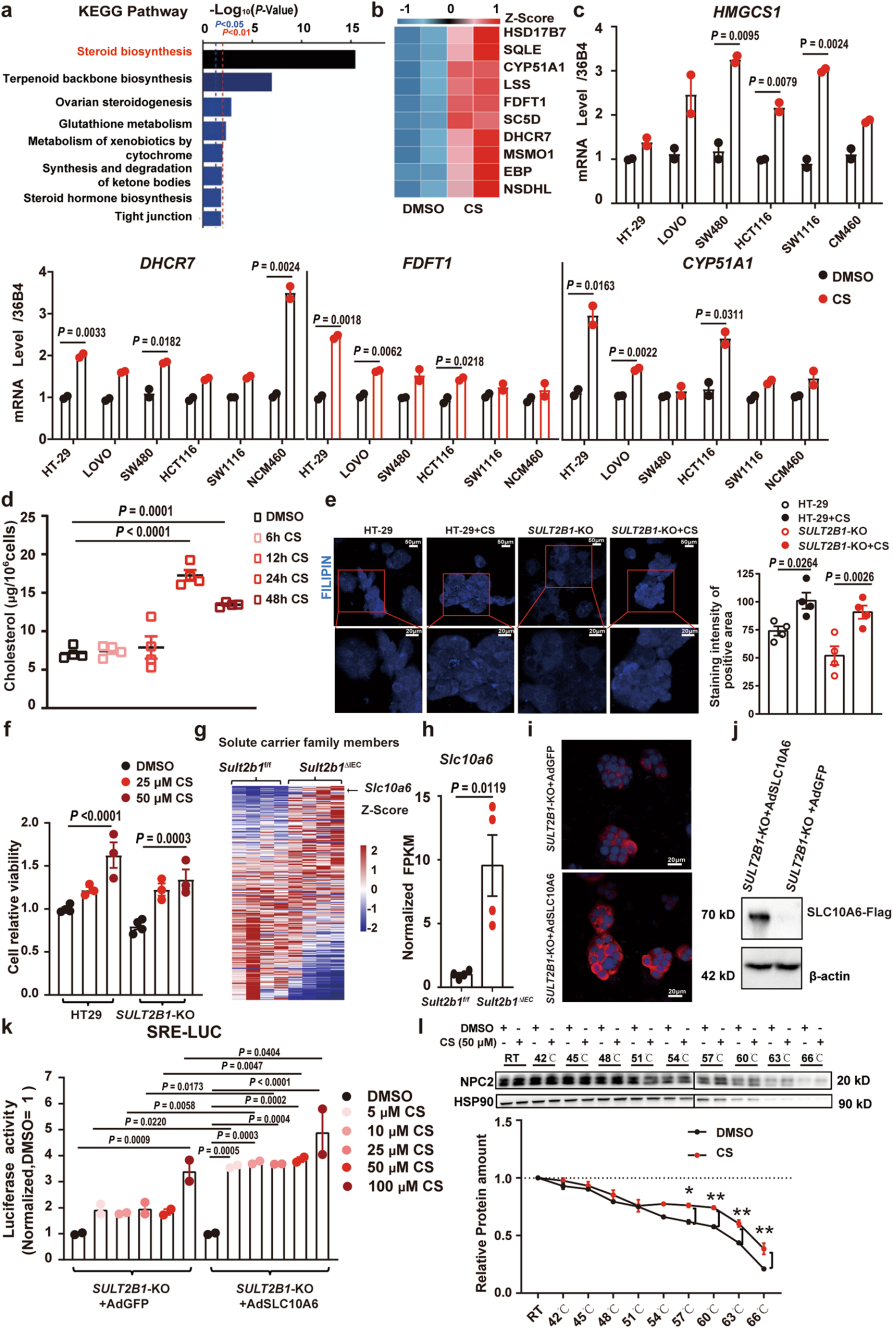
CS promotes cholesterol biosynthesis by binding to NPC2 and activating SREBP2. **a** Kyoto Encyclopedia of Genes and Genomes (KEGG) pathways analysis for the up-regulated differentially expressed genes in CS-treated HT-29 cells compared with control HT-29 cells (treated with DMSO). (Fold change ≥1.5, *P* < 0.05). **b** Heatmaps for the steroid biosynthesis genes in the upregulated genes group in CS-treated HT-29 cells compared with control HT-29 cells (treated with DMSO). **c** RT-qPCR analysis of *HMGCS1*, *DHCR7*, *FDFT1* and *CYP51A1* mRNA levels in HT-29 cells, LOVO cells, SW480 cells, HCT116 cells, SW1116 cells, and NCM460 cells treated with CS (50 μM) or DMSO for 24 h (*n* = 2 independent culture wells, two-tailed Student’s *t-*test). **d** The cholesterol concentration of HT-29 cells treated with CS (50 μM) for 6, 12, 24, and 48 h measured by the Amplex® Red Cholesterol Assay Kit (*n* = 4 biologically independent samples, one-way ANOVA with Sidak’s multiple comparison test). **e** Filipin III staining in HT-29 and *SULT2B1*-KO HT-29 cells in the presence or absence of CS (50 μM) (*n* = 4 fields/group, one-way ANOVA with Sidak’s multiple comparison test). **f** Cell viability of HT-29 and *SULT2B1*-KO cells treated with 25 μM or 50 μM CS (*n* = 4, 3, 3 biologically independent samples/group, respectively, one-way ANOVA with Sidak’s multiple comparison test). **g** Transcriptional profiles of SLC family members in colonic tissues from *Sult2b1*^f/f^ and *Sult2b1*^∆IEC^ mice challenged with 2.5% DSS (*n* = 4 mice/group). **h** Normalized FPKM of *Slc10a6* in inflamed colonic mucosa based on RNA-seq data of *Sult2b1*^f/f^ and *Sult2b1*^∆IEC^ mice with colitis (*n* = 4 mice/group, two-tailed Student’s *t* test). **i** Immunofluorescent staining of SLC10A6 in *SULT2B1*-KO + Ad-GFP and *SULT2B1*-KO + Ad-SLC10A6 cells, where nuclei were counterstained with DAPI. (*n* = 2 biologically independent samples). **j** Western blotting analysis of SLC10A6-Flag in *SULT2B1*-KO + Ad-GFP and *SULT2B1*-KO + Ad-SLC10A6 cells. (*n* = 4 biologically independent samples). **k** The luciferase activity was measured in *SULT2B1*-KO and *SULT2B1*-KO + Ad-SLC10A6 cells treated with DMSO, and 5 μM, 10 μM, 25 μM, 50 μM, and 100 μM CS, and the results were normalized, with respect to measurements corresponding to DMSO treatment (*n* = 2 biologically independent samples, one-way ANOVA with Sidak’s multiple comparison test). **l** Cellular thermal shift assay of HT-29 cells in the presence or absence of 50 μM CS. Two-way ANOVA with Tukey’s multiple comparisons test, 57 °C (DMSO vs. CS) *P = 0.0316; 60 °C (DMSO vs. CS) **P = 0.0076; 63 °C (DMSO vs. CS) **P = 0.0063; 66 °C (DMSO vs. CS) **P = 0.0044. Data from in vitro assays are reprehensive of at least three independent experiments. Data are shown as the mean ± SEM. Source data are provided as a Source Data file.

To further explore the signaling pathway underlying the mechanism of CS treatment, we treated the HT-29 cells with 50 μM CS for 24 h, and then performed transcriptomic profiling by RNA-seq. CS treatment altered the transcriptomic profile of HT-29 cells (Supplementary Fig. 6a). Using the Kyoto Encyclopedia of Genes and Genomes (KEGG) pathways analysis (Fig. 5a and Supplementary Fig. 6b), steroid biosynthesis pathway was identified as the top significantly upregulated pathway (Fig. 5a). The mRNA levels of genes involved in cholesterol biosynthesis such as *HSD17B7*, *SQLE*, *CYP51A1*, *LSS*, *FDFT1*, *SC5D*, *DHCR7*, *MSMO1*, *EBP*, and *NSDHL* were increased by CS treatment (Fig. 5b). Next, the upregulation of cholesterol biosynthesis genes (*HMGCS1*, *DHCR7*, *FDFT1*, and *CYP51A1*) expression by CS treatment were validated by RT-qPCR in HT-29 cells and other different types of colonic epithelial cells including LOVO, SW480, HCT116, SW1116, and NCM460 cells (Fig. 5c). In addition, in HT-29 cells, CS treatment elevated cholesterol abundance (Fig. 5d). Furthermore, using Filipin III staining, we found that CS increased the level of free cholesterol in both *SULT2B1*-KO and HT-29 wild-type cells and improved cell viability (Fig. 5e, f).

Solute carrier (SLC) family transporters facilitate the transport of a wide array of substrates across biological membranes. More than 300 SLC transporters have been identified to date^34^. We examined the transcriptional profiles of SLC family members in colonic tissues from 2.5% DSS-challenged *Sult2b1*^f/f^ and *Sult2b1*^∆IEC^ mice (Fig. 5g). *Slc10a6* was the top one upregulated gene by *Sult2b1* deletion (Fig. 5g, h). SLC10A6, also known as sodium-dependent organic anion transporter (SOAT), acts as a specific steroid sulfate uptake carrier. Although the ability of SLC10A6 to transport CS in a manner similar to other sulfated steroids has not been clarified, *Slc10a6* knockout mice exhibited elevated serum levels of CS^35^. We used adenovirus (Ad)-SLC10A6 infection to overexpress SLC10A6 in *SULT2B1*-KO HT-29 cells, which was confirmed using immunofluorescent staining and western blotting (Fig. 5i, j). SREBP2 is a key transcription factor for cholesterol biosynthesis. We constructed an SREBP2 response element (SRE)-luciferase reporter, and the luciferase reporter assay revealed that CS enhanced the SRE-luciferase activity, which was further strengthened by overexpression of SLC10A6 in *SULT2B1*-KO HT-29 cells (Fig. 5k).

Previous studies have reported that CS could bind to NPC2 with a higher affinity than cholesterol^28–31^. By binding to NPC2, CS might interfere with cholesterol trafficking, which is critical for SREBP2 activation. To confirm the binding of CS to NPC2 in colonic epithelial cells, we performed a cellular thermal shift assay (CETSA). The assay revealed that CS increased the thermal stability of endogenous NPC2 in HT-29 cells (Fig. 5l). These results suggest that CS is capable of binding to NPC2 in colonic epithelial cells, which might competitively inhibit cholesterol trafficking and subsequently activate SREBP2.

**Fig. 6:**
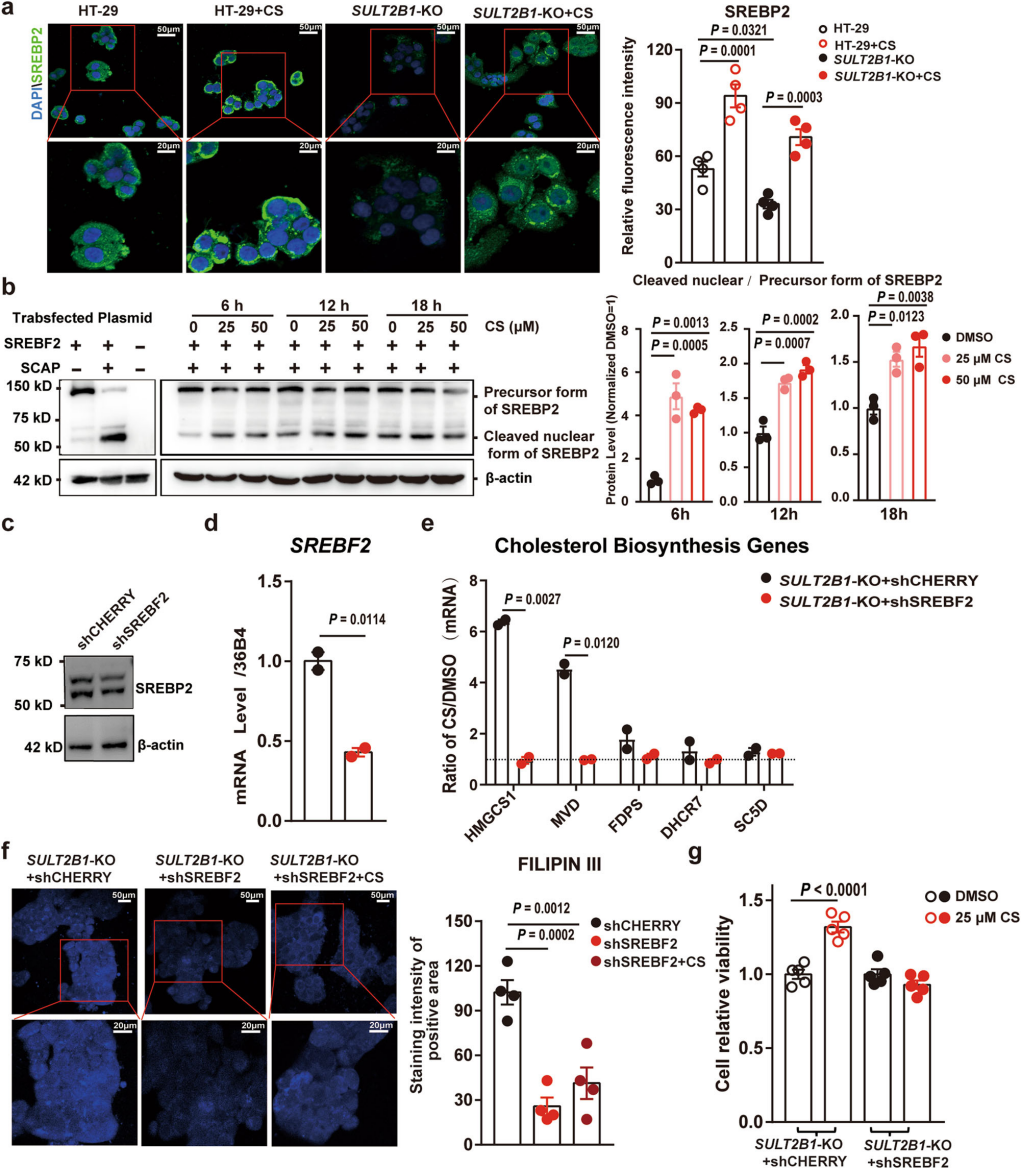
Promotion of cholesterol biosynthesis by CS was dependent on SREBP2. **a** Immunofluorescent staining of SREBP2 in HT-29 and *SULT2B1*-KO cells in the presence or absence of CS (50 μM), where nuclei were counterstained with DAPI (*n* = 4 fields/group, one-way ANOVA with Sidak’s multiple comparison test). **b** Western blotting analysis of mature/precursor SREBP2 in 293T cells transiently transfected pCDH-CMV-HA-SREBF2 /pCDNA3-SCAP and treated with DMSO, 25 μM CS, or 50 μM CS for 6 h, 12 h or 18 h. The ratio of mature/precursor SREBP-2 was summarized. (*n* = 3 biological replicates, one-way ANOVA with Sidak’s multiple comparison test). **c** Western blotting analysis of SREBP2 in *SULT2B1*-KO + sh-CHERRY and *SULT2B1*-KO + sh-SREBF2 cells. **d** RT-qPCR analysis of *SREBF2* in *SULT2B1*-KO + sh-CHERRY and *SULT2B1*-KO + sh-SREBF2 HT-29 cells (n = 2 independent culture wells, two-tailed Student’s *t*-test). **e** RT-qPCR analysis of cholesterol synthesis genes in *SULT2B1*-KO + shCherry and *SULT2B1*-KO + shSREBF2 cells treated with DMSO or CS (50 μM). The values were expressed as CS/DMSO (*n* = 2 independent culture wells, two-tailed Student’s *t*-test). **f** Filipin III staining in *SULT2B1*-KO cells, *SULT2B1*-KO + shSREBF2 cells, and *SULT2B1*-KO + shSREBF2 + CS (50 μM) cells (*n* = 4 fields/group, one-way ANOVA with Sidak’s multiple comparison test). **g** Cell viability of *SULT2B1*-KO and *SULT2B1*-KO + shSREBF2 cells treated with DMSO (served as a control) or 25 μM CS (*n* = 5 biologically independent samples, one-way ANOVA with Sidak’s multiple comparison test). Data are reprehensive of at least three independent experiments. Data are shown as the mean ± SEM. Source data are provided as a Source Data file.

Immunofluorescent staining showed that the expression of mature SREBP2 was decreased by *SULT2B1* deletion but enhanced by CS treatment (Fig. 6a). To confirm that CS promotes SREBP-2 proteolytic activation, we transfected full-length SREBP2 (with HA-tag in NH2-termius) in 293 T cells, and then subjected the whole cell lysates to immunoblot analysis. We detected the precursor form of SREBP2, however, very little of the SREBP2 precursor was processed to the mature form. Referring to a literature published recently^36^, we co-transfected the SREBP-cleavage activating protein (SCAP) which could stabilize full-length SREBP2 and traffic SREBP2 to the Golgi for proteolytic activation; following this, we detected a much higher level of mature SREBP2. Using this system, we observed that CS significantly increased the ratio of mature/precursor SREBP-2 (Fig. 6b), which suggested that CS promoted SREBP2 proteolytic activation. Next, we knocked down SREBP2 expression in *SULT2B1*-KO HT-29 cells using lentivirus-mediated shSREBF2 (Fig. 6c, d) and found that upregulation of the cholesterol synthesis genes *HMGCS1* and *MVD*, caused by addition of CS, was inhibited by the knockdown of SREBP2 (Fig. 6e). SREBP2 knockdown led to a decrease in the free cholesterol content. CS was not capable of promoting free cholesterol content in SREBP2-deficient *SULT2B1*-KO HT-29 cells (Fig. 6f). Moreover, the increase in cell growth induced by CS was also prevented by SREBP2 knockdown (Fig. 6g).

Thus, CS promotes cholesterol biosynthesis by binding to NPC2 and activating SREBP2 in colonic epithelial cells.

### SREBP2 inhibitor betulin suppresses the alleviation effects of CS on DSS-induced colitis in mice

**Fig. 7:**
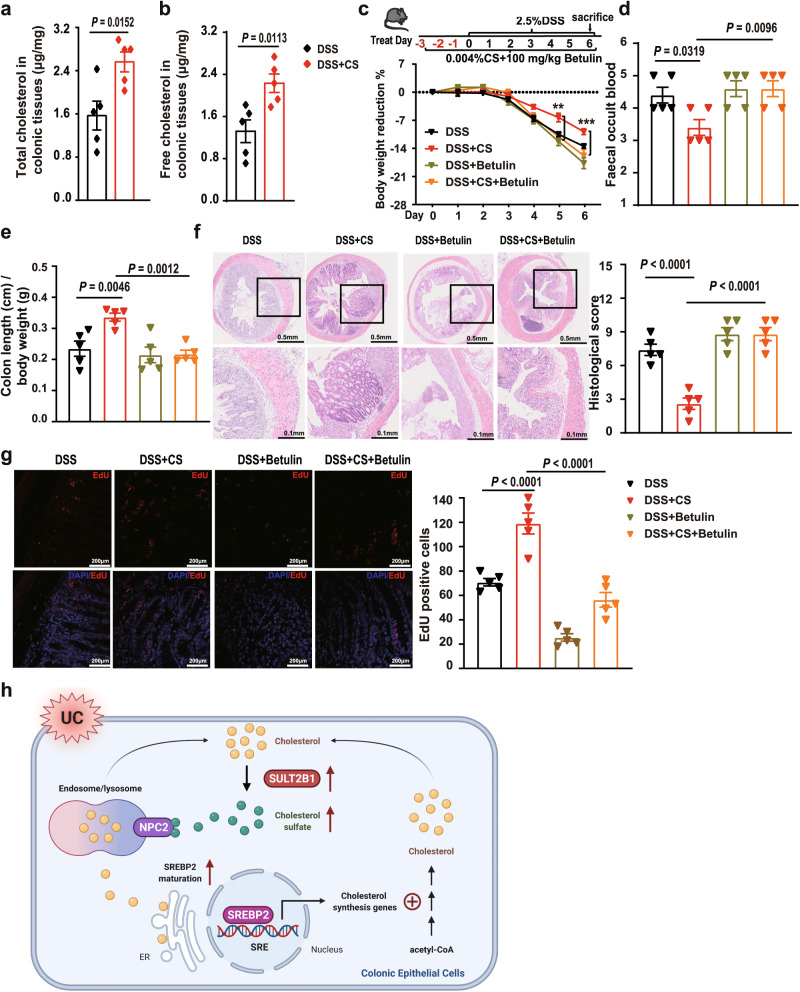
SREBP2 inhibitor betulin suppresses the alleviation effects of CS on DSS-induced colitis in mice. **a**, **b** Total and free cholesterol content of colonic tissues from C57BL/6J mice in the presence or absence of CS (0.004%). (*n* = 5 mice/group, two-tailed Student’s *t*-test). **c** Schematic diagram of treatment and body weight reduction rate in C57BL/6J, 0.004% CS-fed C57BL/6J, C57BL/6J + betulin (100 mg/kg), and 0.004% CS-fed C57BL/6J + betulin (100 mg/kg) mice during DSS challenge (*n* = 5 mice/group). Two-way ANOVA with Tukey’s multiple comparisons test, day 5 (DSS + CS vs. DSS + CS + Betulin) **P = 0.0065; day 6 (DSS + CS vs. DSS + CS + Betulin) ***P < 0.0001. Data are shown as the mean ± SEM. **d**, **e** FOB and colon length/body weight of these mice (n = 5 mice/group, one-way ANOVA with Sidak’s multiple comparison test). **f**, **g** Hematoxylin and eosin and EdU staining of colonic sections excised from the same parts (2 cm away from the anus) of these mice (*n* = 5 mice/group, one-way ANOVA with Sidak’s multiple comparison test). **h** Schematic presentation of the role of CS in intestinal epithelial repairment in colitis. Data are shown as the mean ± SEM. Source data are provided as a Source Data file.

In the DSS-induced colitis model, total and free cholesterol in the colonic tissues were increased by dietary supplementation with CS (Fig. 7a, b), suggesting that dietary CS might ameliorate colitis by increasing cholesterol biosynthesis in the intestine. To test this hypothesis, we introduced the SREBP2 inhibitor, betulin, into DSS-challenged mice by gavage. All the clinical signs of colitis, including body weight loss (Fig. 7c), FOB (Fig. 7d), colon length shortening (Fig. 7e), histological injury (Fig. 7f), and number of EdU-positive cells (Fig. 7g), alleviated by CS were inhibited by betulin. These data suggest that cholesterol biosynthesis in the intestine, controlled by SREBP2 activation, is crucial for the therapeutic effects of CS on colitis in mice.

## Discussion

In the present study, we demonstrated that the levels of CS and cholesterol, as well as the expression of *Sult2b1* and cholesterol biosynthesis genes, were higher in the inflamed tissues from patients with UC than in colonic mucosa from healthy controls. Furthermore, we found that CS in the colonic mucosa, intestinal lumen, and circulation was mainly produced by intestinal epithelial SULT2B1. CS deficiency caused by IEC-specific deletion of *Sult2b1* aggravated DSS-induced colitis in mice, while dietary supplementation with CS ameliorated colitis in acute and chronic UC mice. Mechanistically, CS promoted cholesterol biosynthesis by binding to NPC2 and activating SREBP2 in colonic epithelial cells, thereby alleviating UC in mice (Fig. 7h).

Based on our data, SULT2B1 was predominantly expressed in the gastrointestinal tract. IEC-specific deletion of *Sult2b1* not only leads to deficiency of CS in colonic tissues and cecal contents but also results in a loss of over 90% of CS in circulation, which suggests that intestinal SULT2B1 is responsible for producing abundant CS in the intestinal epithelial cells and lumen. More importantly, abundant CS present in circulation is mainly produced in the gut. Our study elucidates the biological origin of circulatory CS in mice. CS produced from intestinal segments is not a metabolic waste from cholesterol but serves important regulatory functions, particularly during intestinal diseases, such as colonic colitis.

The amount of cholesterol and CS, as well as the mRNA levels of cholesterol biosynthesis genes and *SULT2B1*, in the inflamed tissues from patients with UC were higher than those in colonic mucosa from healthy controls. This phenomenon can be explained by the upregulation of SULT2B1 by proinflammatory factors. On day 6 of the 2.5% DDS-induced colitis mouse model, SULT2B1 and CS levels in colonic epithelium were decreased due to severe colonic epithelium loss. However, both short-term (2.5%) DSS challenge followed by recovery for several days and prolonged low-dose (0.5%) DSS challenge led to enhanced SULT2B1 expression in colonic tissues. In summary, the upregulation of CS and SULT2B1 might be an adaptive and protective response for mucosal healing in UC.

Enterocytes take up cholesterol from plasma lipoproteins, including HDL and LDL, at the basolateral side. The liver and intestinal mucosa are cholesterol synthesis centres, and the majority of cholesterol synthesis occurs in the liver, followed by the intestinal mucosa^9,37^. Corroborating with previous reports^38,39^, our retrospective analysis data (Supplementary Fig. 7a–d, Supplementary table 7) also revealed lower plasma, cholesterol and LDL-C and HDL-C concentrations, as well as higher triglyceride (TG) levels in 113 patients with UC than in 123 healthy controls. Hypocholesterolaemia, found in subjects with UC, might be caused by intestinal malabsorption, malnutrition, or other undetermined mechanisms. Similarly, the serum total and free cholesterol levels were reduced in C57BL/6J mice with DSS-induced colitis (Supplementary Fig. 7e). As *Sult2b1*^∆IEC^ mice exhibited severe colitis following DSS challenge, their serum free cholesterol level was also lower than that of control mice (Supplementary Fig. 7f). Lower HDL-C and LDL-C levels in patients with UC make it difficult for colonic epithelial cells to obtain plasma-supplemented cholesterol. We observed the increase in cell and crypt growth following supplementation with β-cyclodextrin-loaded cholesterol. Without β-cyclodextrin as carriers, both high- and low-dose cholesterol supplementation in diets were not able to ameliorate DSS-induced colonic colitis, which might be related to the absence of intestinal cholesterol absorption gene Npc1l1 expression in colon mucosa (Supplementary Fig. 1h). Thus, under colitis challenge, supplementation of cholesterol in the colonic epithelial cells relies more on cholesterol biosynthesis but not on plasma lipoprotein replenishment or cholesterol uptake from the intestinal lumen.

SREBP2, the master transcriptional regulator of cholesterol biosynthesis, is synthesized in the endoplasmic reticulum (ER). When cholesterol in the ER membrane is depleted, the SCAP-SREBP2 complex moves from the ER to the Golgi for proteolytic activation of SREBP2. The processed SREBP2, designated as mature SREBP2, then enters the nucleus as a homodimer and binds to the sterol regulatory element (SRE) sequence in the promoter of the cholesterol biosynthesis genes and upregulates their transcription^40^. Our in vitro and in vivo data suggested that CS enhanced SREBP2 processing, thereby promoting cholesterol biosynthesis and colonic epithelial cell proliferation. In 2006, using chromatography-based binding assays, Liou et al. reported that CS, among all sterols, had the highest affinity for NPC2^28^. In 2007, Xu et al. reported that the crystal structure of bovine NPC2 bound tightly to CS, with an apparent affinity greater than that of cholesterol, and has the ability to completely displace the previously bound cholesterol from NPC2^27^. In 2008, Infante et al. reported that CS inhibited the binding of [^3^H] cholesterol to NPC2, whereas it did not inhibit the binding to NPC1^29^. Subsequently, the high-affinity binding of CS to NPC2 was further confirmed by other studies^30,31^. CETSA is a powerful method for assessing target engagement by monitoring ligand-induced changes in the thermal stability of cellular proteins. Using CETSA, we demonstrated that treatment with CS increased the thermal stability of endogenous NPC2 in HT-29 cells. These data suggest that CS is capable of binding to NPC2 in colonic epithelial cells. Therefore, binding of CS to NPC2 may affect lysosomal cholesterol transport, which is followed by trafficking of cholesterol to the downstream ER membrane. Changes in ER cholesterol trigger SREBP2 activation and subsequent cholesterol biosynthesis gene expression. Overall, our data suggest that CS may promote de novo cholesterol biosynthesis by binding to NPC2 and competitively inhibiting lysosomal cholesterol transport.

In summary, we elucidated that intestinal epithelial SULT2B1 is most crucial for producing CS in the gut and circulation. Dietary supplementation with CS alleviated DSS-induced colitis, thereby providing a novel strategy for the treatment of experimental UC. Our study reports that CS, an endogenous active cholesterol derivative, contributes to the healing of the mucosal barrier and exhibits remarkable therapeutic efficacy for UC in mice.

## Methods

### Animal studies

All animal studies were performed according to protocols approved by the Animal Ethics Committee of the Fudan University School of Basic Medical Sciences. Age- and sex-matched, 8–12-week-old C57BL/6J mice used in this study were purchased from the Experimental Animal Center of Shanghai, SLAC. CRISPR/Cas9 technology at GemPharmatech Co., Ltd. (Nanjing, China) was used to modify the *Sult2b1* gene. Briefly, sgRNA targeting exon 3 of the *Sult2b1* gene was transcribed in vitro, and a donor vector was constructed. Cas9, sgRNA, and donor were microinjected into fertilized eggs of C57BL/6JGpt mice. Fertilized eggs were transplanted to obtain F0-positive mice, which were confirmed using PCR and sequencing. A stable F1 generation mouse model was obtained by mating positive F0 generation mice with C57BL/6JGpt mice. The floxed mice were knocked out after mating with mice expressing Villin-Cre recombinase, resulting in the loss of function of SULT2B1 in intestinal epithelial cells. *Sult2b1*^f/f^ Villin-Cre (*Sult2b1*^∆IEC^), and littermate control *Sult2b1*^f/f^ mice were generated by crossing Cre-positive and Cre-negative mice. All mice were fed in laboratory animal facilities, given free access to food and water, under a controlled room temperature (22 ± 1 °C) and humidity (65 ± 5%) with a standard 12 h light-dark cycle. To establish DSS-induced colitis, mice received 2.5% (2.5 g/100 mL) DSS (MP Biomedicals, USA; MW: 36,000–50,000 Da, Cat. #160110) for six consecutive days in drinking water. The body weights of the mice were recorded every day. The FOB was tested on days 3 and 5 using a commercial kit (BASO, Zhuhai, China, Cat. #BA2020B), and colon length was determined at the end of the experiment. The mice were fed a diet supplemented with 0.004% CS or 0.005% cholesterol (Supplementary tables 3 and 4) for three days before DSS treatment and for the following six-day DSS challenge. The mice were administered the SREBP2 inhibitor betulin (100 mg/kg) (MedChemExpress LLC, MONMOUTH Junction, NJ, USA, Cat. # 473-98-3) via oral gavage for ten consecutive days, in addition to CS treatment. The control mice were administered vehicle. Male C57BL/6 interleukin-10 deficient (IL-10^−/−^) mice were obtained from GemPharmatech Co., Ltd. (Nanjing, China). IL-10^−/−^ mice were born and raised in SPF facility until the day they were transferred to our clean facility (age 8–10 week).

### Human biopsy collection

Following informed consent, biopsies were collected from volunteers attending endoscopy for routine colonoscopic screening (healthy) or as part of ongoing clinical care (patients with UC). We used tissues derived from patients with a proven histological diagnosis. In the immunochemical staining experiment, tissues were sampled from clinically inflamed colon and clinically non-involved regions of the same patients with UC. All patients gave informed consents for collection of tissue collection. All procedures were performed in accordance with institutional guidelines and were approved by Shanxi Provincial People’s Hospital Research Ethics Committee with the reference number 2019-70 for this study. The study was conducted in accordance with the criteria set by the Declaration of Helsinki.

### Quantification of cholesterol sulfate by LC–MS

Cholesterol sulfate-d7 (CS-d7, Cat. #IR20993, ISOREAG; Shanghai ZZBio Co., Ltd.), as the internal standard (IS), was prepared at a concentration of 60 ng/mL in methanol. Stock standard of 20 mg of cholesterol sulfate (Cat. #700016P; Sigma-Aldrich) was dissolved in methanol to a volume of 100 mL as a storage solution (200 μg/mL). The CS storage solution was diluted in methanol to 2000, 1000, 400, 200, 100, 40, 20, 10, 4, 2, 1, and 0.4 ng/mL, and then mixed with the IS (the volume ratio was 1:1) to 1000, 500, 200, 100, 50, 20, 10, 5, 2, 1, 0.5, and 0.2 ng/mL, for generating a standard curve.

One hundred microliters of serum sample was mixed with 100 μL of IS (1.5 μg/mL). Protein precipitation occurred after adding 300 μL of methanol and vortexing for 1 min. Fifty milligrams of cecal content sample or colon tissue sample was homogenized in 300 μL of methanol and 200 μL of IS (1.5 μg/mL). After centrifugation at 14,000 *g* for 10 min, the upper phase was transferred to a new tube and diluted with methanol. LC-MS was used to quantitate the extracted cholesterol sulfate in this study by PANOMIX Biomedical Tech Co., LTD (Suzhou, China). Chromatography was performed on an Agilent Poroshell 120EC-C8, 2.1 mm × 100 mm, 1.9 μm HPLC column at 40 °C and subjected to gradient elution. The flow rate was 0.3 mL/min and the injection volume was 5 μL. The mobile phase consisted of solvent A (water with 5 mM formic acid) and solvent B (acetonitrile). The percentage of mobile phase B was 5% in the first 1 min, changed linearly from 5% to 95% over 3 min, maintained for 2 min, then linearly returned to 5% over 10 s, and maintained for 2.9 min. After separation, eluting compounds were ionized using ESI in the negative ion mode. The ionization source parameters were as follows: ion source temperature, 500°C; ion source voltage, −4500 V; collision gas, 6 psi; air curtain gas, 30 psi; atomized gas, 50 psi; auxiliary gas, 50 psi. Multiple reaction monitoring (MRM) scanning was performed. The ion pair used for CS quantitative analysis was 465/97; DP: −40, EP: −10, CE: −49, and CXP: −1. The ion pair used for CS-d7 quantitative analysis was 472/97; DP: −40, EP: −10, CE: −47, and CXP: −3. The linear regression equation was *y* = 0.028 * *x* + 0.0402; *r* = 0.9950. “*x*” denotes the CS concentration. “*y*” denotes the peak area ratio of CS versus CS-d7. The linear range was 0.5–1000 ng/mL and the limit of quantitation was 0.5 ng/mL.

### DESI-MSI experiment

A commercial desorption electrospray ionization (DESI) source (Prosolia, Indianapolis, U.S.) coupled to an LTQ Orbitrap Elite mass spectrometer (Thermo Fisher Scientific, San Jose, CA) was used for mass spectrometry imaging (MSI). The main parameters of DESI-MSI were as follows: acetonitrile-water (7:3, v/v) was constructed as the spray solvent system with the flow rate set at 2.0 μL/min. Nitrogen (1.0 MPa) was used for the nebulizing gas. A high voltage of −4 kV was applied onto the sprayer head to form the charged microdroplets. Given these conditions above, the sprayed droplets spot on the glass slide as well as corresponding lateral resolution can reach to approximate 200 μm. The impact angle and distance between the sprayer and substrate were 55° and 4.0 mm, respectively. The distance between the sprayer tip and the transport tube (coupled to the MS inlet) was 4.5 mm. The MS capillary temperature was set at 275 °C, and the S lens voltage was set at 55 V. The automatic gain control was set at 3E6 and maximum injection time set at 400 ms. By preliminary investigation, the DESI scan rate can be kept constant under these orbitrap parameter settings. DESI-MSI was performed in the negative ion mode within the range of *m/z* 400-500 that covered the deprotonated cholesterol sulfate ion at *m/z* 465.3044 [M-H]^−^ (molecular formula: C27H46O4S). To guarantee the correction of the peak assignment above, the CID-MS/MS experiment was also conducted with the collision energy set at 15 V. Mouse colonic tissues were opened longitudinally, rolled into Swiss rolls and embedded in OCT. After thawing mounted on glass slides, tissue cryosections (10 μm) were fully dehydrated at room temperature before analysis. To compare the CS contents, a diluted series of CS standard-spiked simulative tissue was prepared to construct the quantitation curve according to previous reports^41,42^. After DESI-MSI data acquisition, Xcalibur (Thermo Scientific) was first employed for converting a batch of raw data files into cdf files. Then, the cdf files were imported into a massimager (Chemmind Technologies Co., Ltd, China) for further ion image reconstruction and CS quantitation. Briefly, the CS ion signal variation was first normalized by the total ion current (TIC, range from m/z 400–500) pixel-by-pixel. After the serial simulated intestine section were scanned under completely same DESI condition, a quantitation curve was constructed by fitting the normalized CS ion intensity with its content in each section. Then, the CS content in the real colonic tissue can be estimated by putting each pixel’s CS ion intensity into the quantitation curve.

### Colon colitis scores

For the colon histological analysis, the middle colon segments were embedded in paraffin, sectioned at 5 μm, and stained with haematoxylin and eosin. The colitis score was determined using a standard histologic colitis score (Supplementary Table 5).

### Qualification of cholesterol

Extraction of cholesterol from colonic tissues of patients or mice was performed as described in the above extraction method used for CS measurement. The cholesterol qualification of these extracts as well as of mouse serum was carried out using an Amplex® Red Cholesterol Assay Kit (Invitrogen, Carlsbad, CA, USA Cat. # A12216).

### Preparation of the cholesterol solution used in the in vitro experiments

β-cyclodextrin (0.4 g) was dissolved in 1 mL of physiological saline solution and shaken for 30 min at 37 °C. 11.58 mg cholesterol dissolved in 200 μL CHCL_3_ was dried by nitrogen. The dried cholesterol was dissolved in 1 mL of the above 40% β-cyclodextrin solution to form cholesterol stock solution (30 mM) and shaken overnight at 37 °C. The cholesterol stock solution was shaken for at least 2 h at 37 °C before use.

### Cell lines

HEK293T cell line, human colon carcinoma cell lines HT-29, LOVO, SW480, HCT116, and SW1116 were from National Collection of Authenticated Cell Cultures (Shanghai, China, Cat. #SCSP-502, #SCSP-5032, #SCSP-514, #SCSP-5033, #TCHu99, #TCHu174, respectively).

### Construction of the *SULT2B1*^−/−^ HT-29 cell line

To construct the *SULT2B1* knockout HT-29 cell line, an online sgRNA design tool based on the clustered, regularly interspaced, short palindromic repeats (CRISPR)/CRISPR-associated protein 9 (CAS9) (CRISPR/CAS9) system (http://crispr.mit.edu/) was utilized. Two sgRNA oligos targeting exon 2 of the *SULT2B1* gene were selected as follows: 5′-CGAGTACAGGCCGACGGGGAAGG-3′ (sgRNA-A) and 5′-CGGAGAACACCCAAGATGTGCGG-3′ (sgRNA-B). sgRNA-A and sgRNA-B were cloned into the pSpCas9n(BB)−2A-Puro vector (pX462 vector, Addgene plasmid # 48141). Then, the constructed plasmids containing sgRNA-A and sgRNA-B were co-transfected into HT-29 cells with Lipofectamine 3000 (Invitrogen, CA, USA). *SULT2B1* knockout was confirmed by western blotting and gene sequencing.

### RNA sequencing (RNA-seq)

RNA purified from tissues and cells as indicated was converted into cDNA libraries using the TrueLib mRNA library prep kit for Illumina (ExCell. Bio, China). The library quality was assessed using an Agilent 2100 Bioanalyzer. Sequencing was performed on an Illumina HiSeq X Ten sequencer at Annoroad Gene Technology Co., Ltd. (Beijing, China).

### EdU staining

Mice were injected with EdU (5 mg/kg) intraperitoneally 6 h before sacrifice. The colon tissues were embedded in paraffin for the subsequent staining. Cell proliferation was assessed by the Cell-Light EdU Apollo 567 In Vitro Kit (RiboBio, Guangzhou, Guangdong, China. Cat. #C00003), according to the manufacturer’s instructions. In brief, the tissue was permeabilized with 0.5% Triton X-100 and reacted with 1 × Apollo reaction cocktail for 30 min. Subsequently, the DNA contents of the cells were stained with DAPI for 30 min and visualized under a fluorescence microscope (Leica Microsystems GmbH, DMI6000B).

### FILIPIN staining

Cells were seeded on coverslips placed in 24-well plates. After additional cholesterol or cholesterol sulfate treatment, cells were washed with PBS 3 times and fixed in 4% paraformaldehyde for 20 min at room temperature. Cells were then incubated with 50 μg/mL filipin III (Thermo Fisher Scientific) for 1 h at room temperature. The staining was examined with ultraviolet excitation using a confocal microscope (ZEISS, Germany, LSM 7 10).

### RNA extraction and RT-qPCR

Total RNA was extracted from cells by TRIzol reagent (Invitrogen, Carlsbad, CA, USA Cat. #12183555) following the manufacturer’s instructions and used to generate cDNA using the ReverTra Ace qPCR RT Kit (TOYOBO, Cat. #QPK-201). SYBR green (Yeasen, Shanghai, China, Cat. #11203ES03)-based qPCR was performed with the primers listed in Supplementary Table 6.

### Western blot

Different samples were lysed in RIPA buffer containing a protease and phosphatase inhibitor cocktail (Bimake, Cat. # 510026, Cat. # 410043). The protein concentrations were detected using a BCA protein quantification kit (Biocolor, Cat. # K3000). Then, the same amounts of protein were separated by SDS-PAGE and transferred onto PVDF membranes. The membranes were blocked with 3% BSA in TBST and probed with anti-human SREBP2 (ABclonal, Cat. #A13049, 1:1000), anti-human SULT2B1 (R&D Systems, Cat. #AF6174, 1:1000), anti-mouse SULT2B1 (Santa Cruz Biotechnology, Cat. #sc-166423, 1:1000), anti-human NPC2 (Proteintech, Cat. #19888-1-AP, 1:1000), Monoclonal ANTI-FLAG® M2 antibody (Sigma-Aldrich, Cat. #F1804, 1:2000), HA-tag rabbit mAb (Cell Signaling Technology, #3724), and anti-β-actin (Proteintech, Cat. #66009-1, 1:2000) or anti-HSP90 (Proteintech, Cat. #60318-1-Ig, 1:2000) for the loading control. Then, the membranes were washed with PBST and incubated with peroxidase-conjugated goat anti-rabbit secondary antibodies (Yeasen, Cat. #33101ES60, 1:2500), peroxidase-conjugated goat anti-mouse secondary antibodies (Yeasen, Cat. #33201ES60, 1:2500) or peroxidase-conjugated rabbit anti-sheep secondary antibodies (Abcam, Cat. #ab6747, 1:2500). Immunoreactivity was observed with an ECL Western Blotting Substrate kit (Tanon, Shanghai, China, Cat. #180-501) using a gel imaging system (Tanon-4200, Shanghai, China).

### Immunofluorescence staining

The cells were seeded on coverslips and treated as indicated. The slides were incubated with anti-human SREBP2 (ABclonal, Cat. #A13049, 1:200) or anti-human SLC10A6(Invitrogen, Cat. PA5-53468, 1:200) at 4 °C overnight and then incubated with a fluorescent secondary antibody at room temperature for 90 min. The nuclei were visualized using DAPI. Representative images were acquired with a fluorescence microscope (Leica Microsystems GmbH, DMI6000B).

### Immunohistochemistry staining

Human biopsies were embedded in paraffin. Sections were incubated with primary antibodies, namely, anti-human SULT2B1 (R&D Systems, Cat. #AF6174, 1:100) at 4 °C overnight, followed by incubation with a peroxidase-conjugated rabbit anti-Sheep secondary antibody (Abcam, Cat. #ab6747, 1:500). at room temperature for 90 min, visualization with DAB, and counterstaining with haematoxylin. The H-SCORE calculated by multiplying the quantity and intensity scores was used to semi-quantify SULT2B1 staining for each tissue sample. Scoring criteria for quantity (the estimated proportion of staining in intestinal epithelial cells that was positively stained) were as follows: score = 0, none; score = 1, 1–25%; score = 2, 26–50%; score = 3, 51–75%; and score = 4, 76–100%. An intensity score represented the average intensity of the positive cells, as follows: 0 (none); 1 (weak); 2 (intermediate); and 3 (strong). The proportion and intensity scores were then multiplied to obtain a total score, which could range from 0 to 12. Each sample was evaluated in a blinded manner by two senior pathologists, and conflicting cases were reanalysed by a third pathologist.

### Generation of a stable SREBP2 knockdown HT-29 cell line

The small hairpin RNA sequence against human *SREBF2* was obtained from Sigma-Aldrich. The target shRNA sequence was 5′‐CCGGCCTCAGATCATCAAGACAGATCTCGAGATCTGTCTTGATGATCTGAGGTTTTT‐3′. It was cloned into the mCherry PTSB-SH-mCherry-2A-NEO vector. Lentivirus‐encoded shRNA against human *SREBF2* and the control were prepared. Lentiviral particles were generated through transient transfection of retroviral vectors into the 293T packaging cell line. Supernatants were harvested two days after transfection. HT-29 cells were infected with lentiviral particles and selected against G418. Successful knockdown was confirmed by RT-qPCR and western blot analysis.

### Luciferase Reporter Assay

The 6 × SREBP2 response element (SRE) consensus sequence (ATCACCCCAC- ATCACGCCAC-GTCACCCCAT-ATCACCCCAC-ATCACGCCAC-GTCACCCCAT) were cloned into the pCDH-NC-EF1-PURO-F2A-RLUC Lenti-vector. HEK293T cells were cotransfected with a lentiviral plasmid system containing SRE-Reporter-LUC or control-LUC as transfer vector, PMD2.G, and PxPSA2.0 as packaging vectors. The lentivirus-containing supernatants were collected, filtered at 48 h and 72 h after transfection. HT-29 cells were infected with the respective lentivirus. The infected cells were seeded in 24-well plates, and then treated with CS or DMSO. After 24 h, Cells were lysed in passive lysis buffer (PLB). Luciferase activity was measured by using a luciferase reporter assay (Promega, Cat. #E1960).

### Transient transfection of cells

pCDH-CMV-HA-SREBF2 is an expression plasmid that encodes, in sequential order from the NH2-termius, a HA epitope tag (YPYDVPDYA) and human full-length SREBF2. pCDNA3-SCAP is an expression plasmid that encodes human full-length SCAP. The above plasmids were generated by standard cloning methods. All plasmids were verified by sequencing the entire coding region. 1.5 µg pCDH-CMV-HA-SREBF2 and 0.75 µg pCDNA3-SCAP were transiently co-transfected into 293T cells cultured in 6-well plates using polyethylenimine (PEI). After 6 h, the serum-free medium was changed to fresh complete medium. After 24 h, CS in the final concentration 25 μM or 50 μM was added to the medium. After further incubation for 6, 12, or 18 h, cells were harvested, and equal fractions of whole cell lysates were subjected to western-blot analysis of SREBP2 (anti-HA) and β-actin.

### Cellular Thermal Shift Assay (CETSA)

For a CETSA in living HT-29 cells, cells were seeded in 60 mm dishes and exposed to CS or DMSO at the indicated concentrations for 1 h in the cell culture incubator. Following incubation, the cells were washed with PBS and harvested in 500 μL of PBS supplemented with complete protease inhibitor cocktail. The cell suspensions were freeze-thawed three times using lipid nitrogen. The lysates were centrifuged at 20,000 x *g* for 20 min at 4 °C. The supernatants were transferred to new tubes. For a CETSA, the cell lysates were divided into smaller aliquots and heated individually at different temperatures for 3 min followed by cooling for 3 min at room temperature. In order to separate the soluble fractions from precipitates, the heated lysates were centrifuged at 20,000 x g for 20 min at 4 °C. The supernatants were transferred to new microtubes and analyzed by SDS-PAGE followed by western blot analysis.

### Statistical analyses

Data are presented as the mean ± standard error of the mean (SEM). GraphPad Prism 8.0 Software (San Diego, CA, USA) was used for calculations, statistical analyses, and graphic generations. Statistical analyses comparing two parameters (between treatments or genotypes) were conducted using the two-tailed Student’s *t*-test. Statistics for multiparameter analyses were determined by one-way analysis of variance (ANOVA) followed by the recommended post hoc tests in GraphPad Prism 8.0 Software. Two-way analysis of variance (ANOVA) was used for time-series experiments. For in vitro assays, experiments were repeated at least three times. *P* < 0.05 was considered significant.

### Reporting summary

Further information on research design is available in the Nature Research Reporting Summary linked to this article.

## Supplementary information

Supplementary Information file

Reporting Summary

## Data Availability

The transcriptional expressions of mouse *Npc1l1* and human *SULT2B1*, *HMGCS1*, *MVK*, *MVD*, *FDPS*, *FDFT1*, *SQLE*, *LSS*, *CYP51A1, MSMO1*, *NSDHL, SC5D*, and *DHCR7* were analyzed from the published GEO datasets (http://www.ncbi.nlm.nih.gov/geo/). The gene expression profiles of the mouse jejunum, ileum, and colon were obtained from GEO (GSE 143342). The RNA-seq data from patients with clinical UC were from GEO (GSE 111889). Raw data of RNA sequencing generated in this study were deposited at SRA database of NCBI with the accession number PRJNA644070. If needed, contact X.L. for original data described in the paper. Contact X.L. for requesting *Sult2b1*^f/f^ and *Sult2b1*^∆IEC^ mouse strain, and all other plasmids or reagents described in this article. Source data are provided with this paper.

## Source data

Source Data

## Code availability

The gene ontology (GO) and Kyoto Encyclopedia of Genes and Genomes (KEGG) database used in the study are available at http://geneontology.org/ and https://www.genome.jp/kegg/. Gene set enrichment analysis (GSEA) was performed using the GSEA software (http://software.broadinstitute.org/gsea/index.jsp).
