# Supplementary material for: Cholesterol sulfate alleviates ulcerative colitis by promoting cholesterol biosynthesis in colonic epithelial cells
Source: Nat Commun. 2022 Jul 30;13:4428. doi: 10.1038/s41467-022-32158-7 (PMC9338998; doi:10.1038/s41467-022-32158-7)
Supplement: Supplementary file 1 — Supplementary Information file [file 41467_2022_32158_MOESM1_ESM.pdf]

Supplementary information

Supplementary Methods

Isolation of intestinal LPMCs and flow cytometry

The colon was removed from the sacrificed mice, cut into 0.5-cm pieces and washed thoroughly with cold PBS to remove the remaining faeces. The pieces were incubated with 2 mM dithiothreitol (DTT) and 1 mM EDTA at 37°C for 45 min with gentle shaking to remove intestinal epithelial cells (IECs). Then, the pieces were digested with collagenase A (1 mg/mL) at 37°C with shaking (150 rpm) for 30 min. The digested products were then collected and further purified by density gradient centrifugation with 40% and 70% Percoll–RPMI solution, and finally, the intestinal lamina propria mononuclear cells (LPMCs) were collected from the interphase. Then, flow cytometric analysis was performed. For surface staining, B cells were gated as $CD45^+$ (clone: 30-F11, Invitrogen, Cat. #11-0451-82, 1:100) and $CD19^+$ (clone: eBio1D3 (1D3), Invitrogen, Cat. #17-0193-80, 1:100) cells. Total T cells were gated as $CD45^+$ and $CD3e^+$ (clone: 145-2C11, Invitrogen, Cat. #17-0031-81, 1:100). $CD4^+$ T cells were gated as $CD45^+$ and $CD4^+$ (clone: GK1.5, Invitrogen, Cat. #12-0041-81, 1:100) cells. $CD8^+$ T cells were gated as $CD45^+$ and $CD8a^+$ (clone: 53-6.7, Invitrogen, Cat. #25-0081-81, 1:100) cells, neutrophils were gated as $CD45^+$ and $Ly6G^+$ (clone: RB6-8C5, Invitrogen, Cat. #25-5931-81, 1:100) cells, and macrophages were gated as $CD45^+$ and $CD11b^+$ (clone: M1/70, Invitrogen, Cat. #17-0112-81, 1:100) cells.

Crypt isolation and intestinal organoid culture

Intestinal crypt cultures were derived from 6-week-old male *Sult2b1*$^{\Delta IEC}$ and *Sult2b1*$^{f/f}$ mice. The colon was isolated, opened longitudinally and flushed gently with ice-cold PBS 3-5 times. Then, the intestinal villi were removed by a glass scraper, and the intestine was cut into 2-mm pieces. The pieces were washed with ice-cold PBS 15-20 times and incubated in 2 mM EDTA for 30 min at 4°C. The suspension was passed through a 70-μm cell strainer, and crypt fractions were isolated and purified through centrifugation for plating in Matrigel. Intestinal organoids were grown in Complete IntestiCult™ Organoid Growth Medium (STEMCELL, Cat. #06005).

Retrospective analysis of cases

Electronic medical charts of patients treated in Shanxi Provincial People's Hospital between 2018 and 2019 were reviewed retrospectively for the diagnosis of UC. The

diagnosis of UC was confirmed after review of admission notes and diagnostic studies, including endoscopy and pathology reports. A total of 113 patients with a confirmed diagnosis of UC, recorded measurements of lipoprotein lipids (complete or partial lipoprotein lipids record) and who were not currently prescribed statin therapy were included in the study. A total of 123 healthy people undergoing health examinations in the outpatient department were selected as healthy controls. The demographic and clinical characteristics of these subjects are showed in Supplementary Table 7.

Supplementary Figures

Supplementary Figure 1

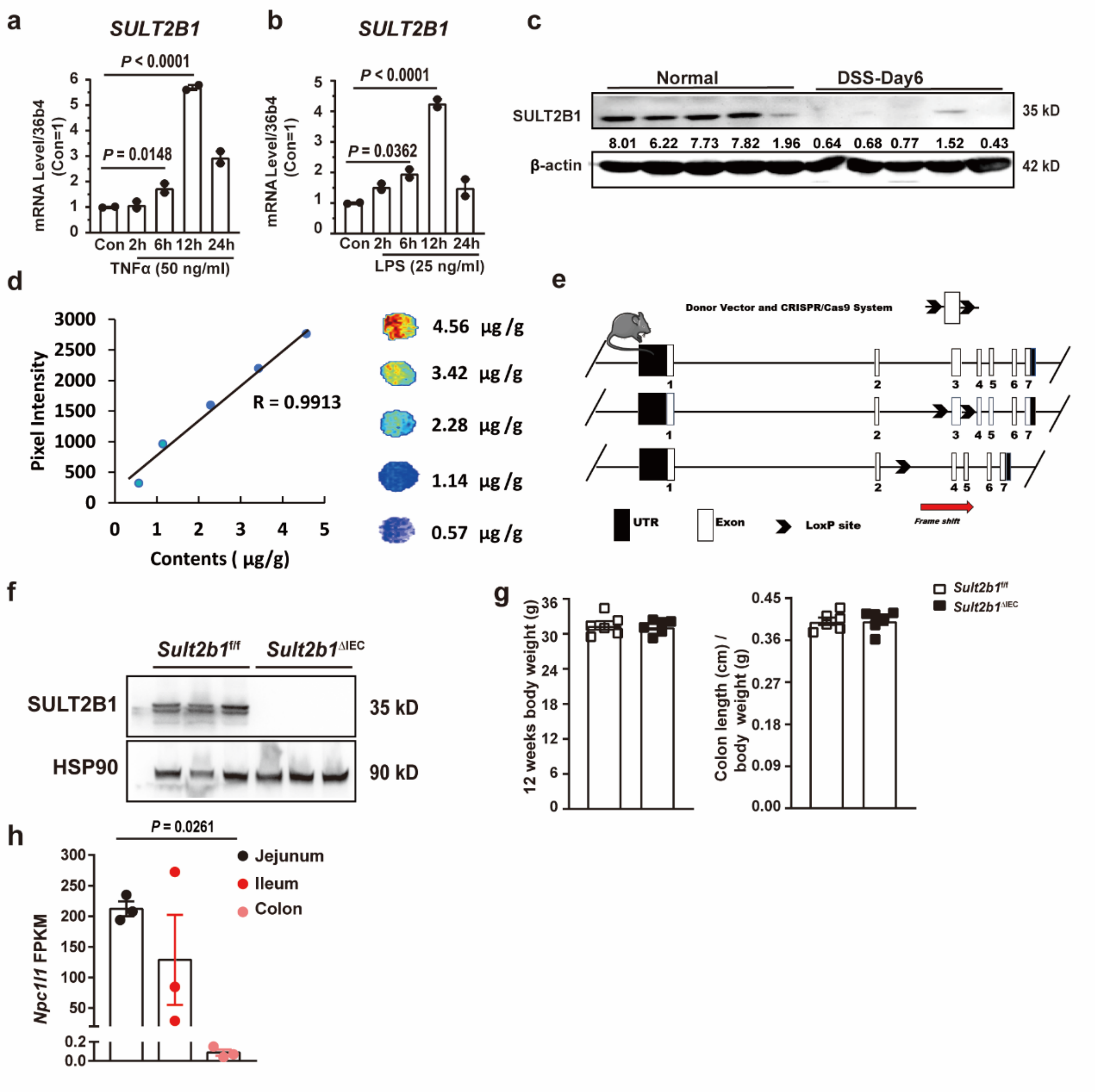

Supplementary Figure 1 a: RT-qPCR analysis of *SULT2B1* mRNA in HT-29 cells treated with TNFα (50 ng/mL) for 2, 6, 12 and 24 h (n = 2 independent culture wells). **b:** RT-qPCR analysis of *SULT2B1* mRNA in HT-29 cells treated with LPS (25 ng/mL) for 2, 6, 12 and 24 h (n = 2 independent culture wells). **c:** Western blotting analyses were performed of SULT2B1 protein in the colonic tissue from Normal C57BL/6J and C57BL/6J mice with 6-day 2.5%DSS challenge (n = 5 mice/group). **d**: The standard curve of desorption electrospray ionization–mass spectroscopy imaging (DESI–MSI). **e**: Generation of intestinal epithelial cells-specific *Sult2b1* knockout mice. **f**: Western blotting analyses were performed of SULT2B1 protein in the colonic tissue from *Sult2b1*$^{f/f}$ and *Sult2b1*$^{\Delta IEC}$ mice (n = 3 mice/group). **g:** The body weight and colon length/body weight of 12-week *Sult2b1*$^{f/f}$ and *Sult2b1*$^{\Delta IEC}$ mice (n = 6 mice/group). **h:** Normalized FPKM (fragments per kilobase of transcript per million mapped reads) of *Npc1l1* in jejunum, ileum and colon based on RNA-seq data in Gene Expression Omnibus (GSE 143342) (n = 3 mice/group). Statistical significance was determined using one-way ANOVA with Sidak's multiple comparison test (a, b). Data from in vitro assays are reprehensive of at least three independent experiments. Data are shown as the mean ± SEM. Source data are provided as a Source Data file.

Supplementary Figure 2

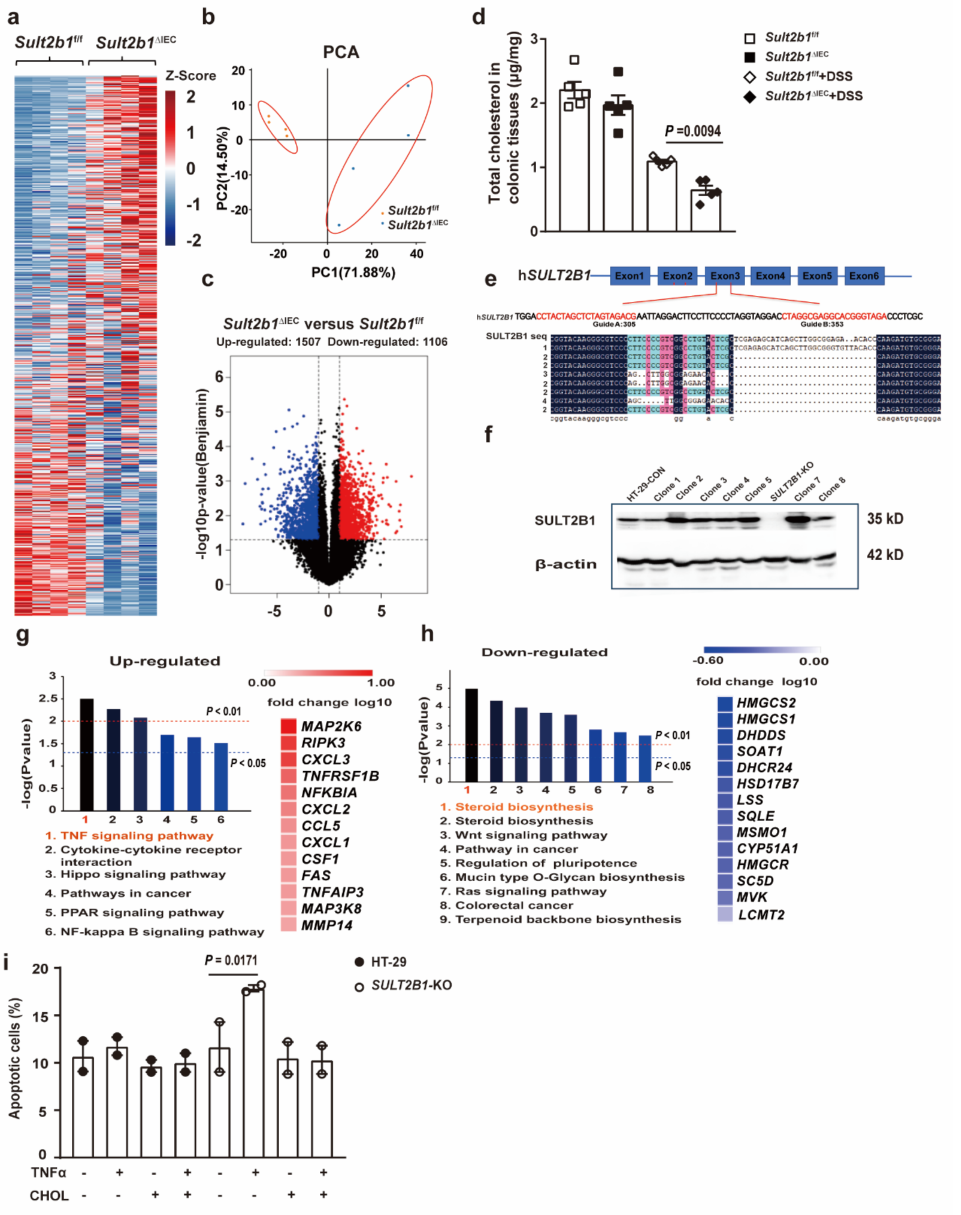

Supplementary Figure 2 a: Transcriptomic profiling of colonic tissue from six-day-DSS-challenged *Sult2b1*$^{f/f}$ and *Sult2b1*$^{\Delta IEC}$ mice was illustrated by Heatmap (n=4 mice/group). **b:** Principal components analysis (PCA) of transcriptomic profiling of colonic tissues from 6-day-DSS-challenged *Sult2b1*$^{f/f}$ and *Sult2b1*$^{\Delta IEC}$ mice (n=4 mice/group). **c:** Volcano plot of the upregulated and downregulated genes from the transcriptomic profiling of colonic tissues from 6-day-DSS-challenged *Sult2b1*$^{f/f}$ and *Sult2b1*$^{\Delta IEC}$ mice (n=4 mice/group). (Fold change ≥ 2, P<0.05, two-tailed Student's t test). **d:** The total cholesterol concentration of colonic tissue from *Sult2b1*$^{f/f}$ and *Sult2b1*$^{\Delta IEC}$ mice in the presence or absence of 6-day DSS using the Amplex® Red Cholesterol Assay Kit (n=5 mice/group). **e:** Gene sequencing was performed to identify the successful knockout of *SULT2B1* in HT-29 cells. **f:** Western blotting was performed to identify the successful knockout of *SULT2B1* in HT-29 cells. **g:** KEGG enrichment for upregulated genes in *SULT2B1*-KO HT-29 cells compared with wild-type HT-29 cells and heatmaps for the inflammation-related genes in the upregulated genes group in *SULT2B1*-KO HT-29 cells compared with wild-type HT-29 cells. **h:** KEGG enrichment for downregulated genes in *SULT2B1*-KO HT-29 cells compared with wild-type HT-29 cells and heatmaps for the cholesterol synthesis related genes in the downregulated genes group in *SULT2B1*-KO HT-29 cells compared with wild-type HT-29 cells. **i:** The flow cytometric analysis with AnnexinV/PI staining showed the effects of TNFα (50 ng/mL) on HT-29 and *SULT2B1*-KO in the presence or absence of cholesterol (50 µM) (n = 2 independent culture wells). Statistical significance was determined using one-way ANOVA with Sidak's multiple comparison test (d, i). Data from in vitro assays are reprehensive of at least three independent experiments. Data are shown as the mean ± SEM. Source data are provided as a Source Data file.

Supplementary Figure 3

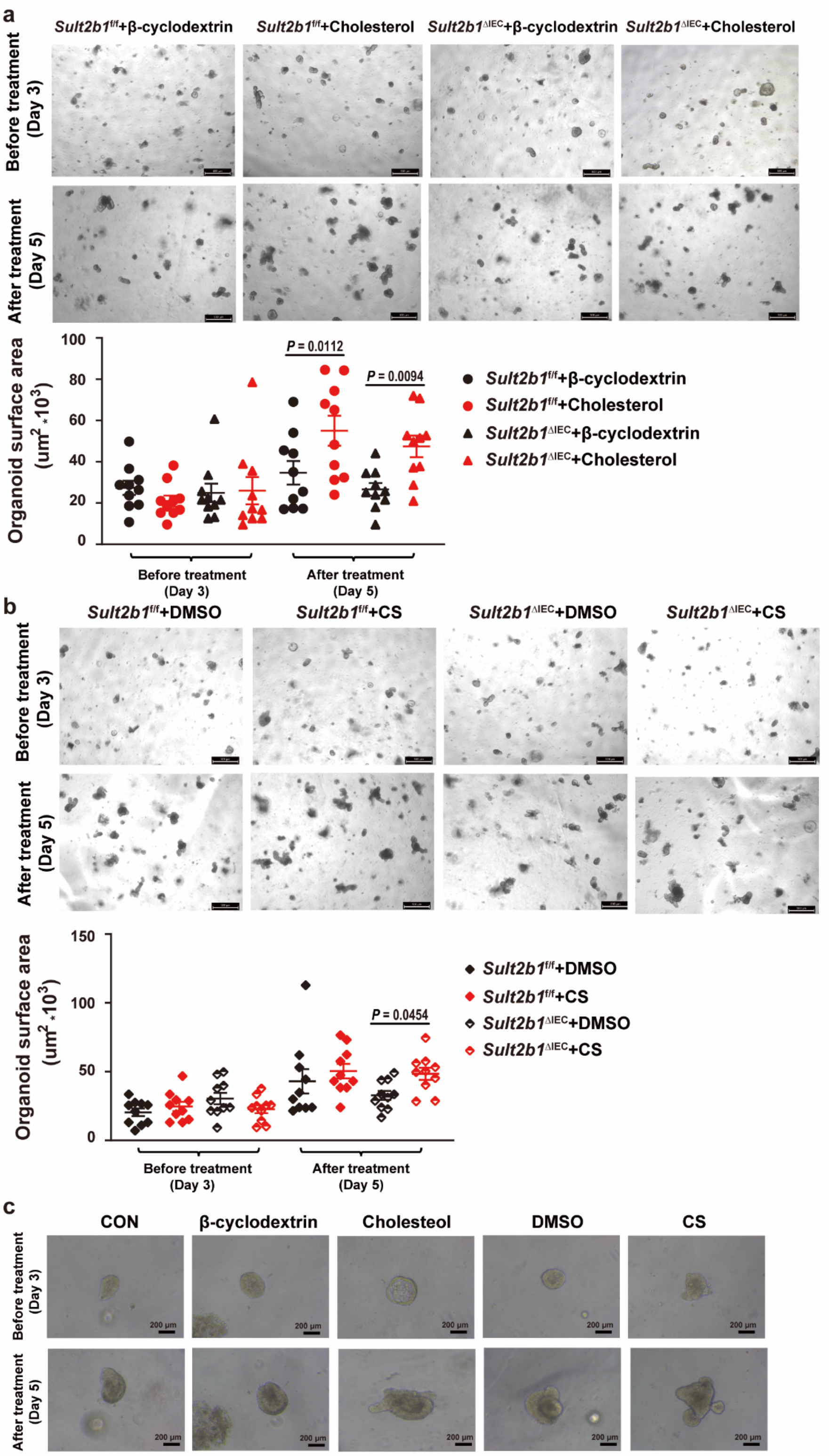

Supplementary Figure 3 a: The area of organoids from *Sult2b1*$^{f/f}$ and *Sult2b1*$^{\Delta IEC}$ mice treated with β-cyclodextrin or 50 μM cholesterol dissolved in β-cyclodextrin (n = 10 biggest single organoids/group). **b**: The area of organoids from *Sult2b1*$^{f/f}$ and *Sult2b1*$^{\Delta IEC}$ mice treated with DMSO or 50 μM CS dissolved in DMSO (n = 10 biggest single organoids/group). **c**: The growth of single representative organoid from *Sult2b1*$^{f/f}$ mice treated with β-cyclodextrin, cholesterol, DMSO or CS. Statistical significance was determined using one-way ANOVA with Sidak's multiple comparison test (a, b). Data are reprehensive of at least three independent experiments. Data are shown as the mean ± SEM. Source data are provided as a Source Data file.

Supplementary Figure 4

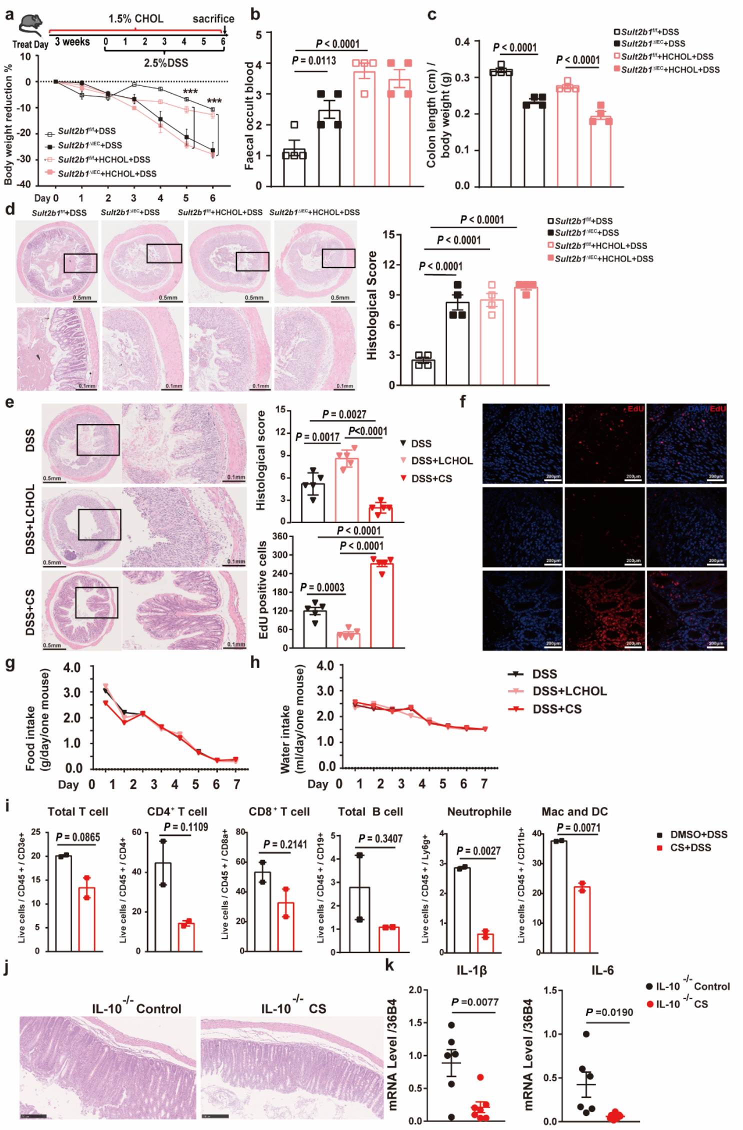

Supplementary Figure 4 a: Schematic diagram of treatment and the body weight reduction rate of *Sult2b1*$^{\Delta IEC}$ and *Sult2b1*$^{f/f}$ mice in the presence or absence of additive cholesterol (1.5% in diet) during the DSS challenge (HCHOL: high dose (1.5%) cholesterol). Two-way ANOVA with Tukey's multiple comparisons test, day 5 (*Sult2b1*$^{f/f}$+HCHOL+DSS vs. *Sult2b1*$^{\Delta IEC}$ +HCHOL+DSS) ***$P < 0.0001$; day 6 (*Sult2b1*$^{f/f}$+HCHOL+DSS vs. *Sult2b1*$^{\Delta IEC}$ +HCHOL+DSS) ***$P < 0.0001$. **b**: Faecal occult blood (FOB) of *Sult2b1*$^{f/f}$ and *Sult2b1*$^{\Delta IEC}$ mice in the presence or absence of additive cholesterol (1.5% in diet) on the day 3 of DSS challenge (n = 4 mice/group). **c**: Colon length/body weight from *Sult2b1*$^{f/f}$ and *Sult2b1*$^{\Delta IEC}$ mice in the presence or absence of additive cholesterol (1.5% in diet) on the day 6 of DSS challenge after their sacrifice (n = 4 mice/group). **d**: Hematoxylin and eosin (HE) staining of colonic tissues (2 cm away from the anus) from 6-day-DSS-challenged *Sult2b1*$^{f/f}$ and *Sult2b1*$^{\Delta IEC}$ mice in the presence or absence of additive cholesterol (1.5% in diet) (n = 4 mice/group). **e-f**: HE and EdU staining of colonic tissues (2 cm away from the anus) from C57BL/6J, LCHOL-fed C57BL/6J, and CS-fed C57BL/6J mice (n = 5 mice/group). (LCHOL: low dose (0.005%) cholesterol). **g-h:** The food and water intake of C57BL/6J, cholesterol-fed (CHOL-fed) C57BL/6J and CS-fed C57BL/6J mice during the DSS challenge (the average value/mouse in one cage). **i:** Quantification of total T cells, $CD4^+$ T cells, $CD8^+$ T cells, total B cells, neutrophils, macrophages, and dendritic cells from the colonic tissues of 6-day DSS-challenged C57BL/6J mice in the presence or absence of CS (0.004% in diet) using flow cytometric analysis (n = 2 mice/group). **j**: HE staining of colonic tissues (2 cm away from the anus) from IL-$10^{-/-}$ mice and CS-fed IL-$10^{-/-}$ mice. **k**: RT-qPCR analysis of IL-1β and IL-6 mRNA levels from the colonic tissues of IL-$10^{-/-}$ mice (n = 6) and CS-fed IL-$10^{-/-}$ mice (n = 7). Statistical significance was determined using one-way ANOVA with Sidak's multiple comparison test (b, c, d, e) or two-tailed Student's t test (i, k). Data from in vitro assays are reprehensive of at least three independent experiments. Data are shown as the mean ± SEM. Source data are provided as a Source Data file.

Supplementary Figure 5

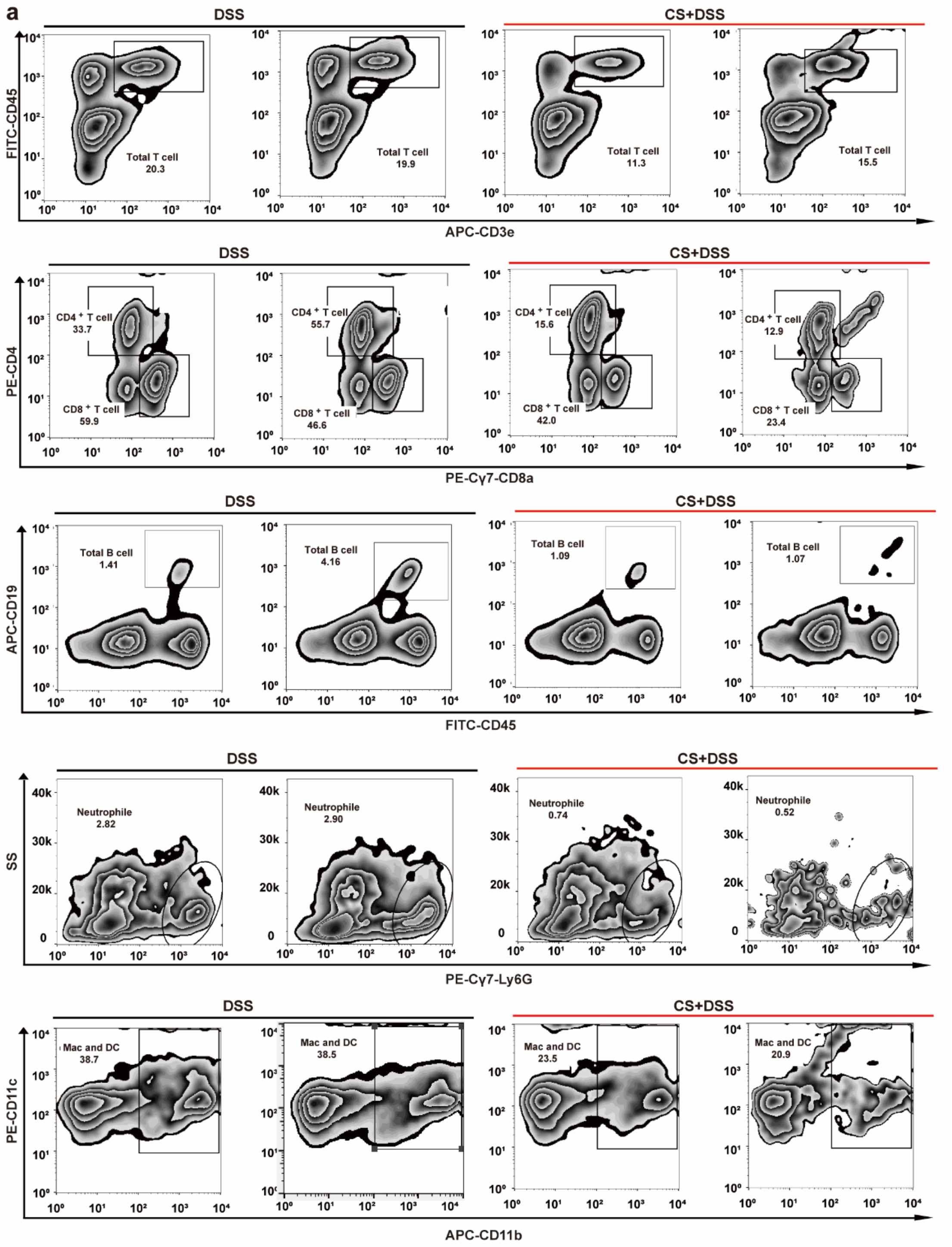

Supplementary Figure 5 a: The gating strategy used to identify total T cells, CD4+ T cells, CD8+ T cells, total B cells, neutrophils, macrophages, and dendritic cells from the colonic tissues of 6-day DSS-challenged C57BL/6J mice in the presence or absence of CS (0.004% in diet) using flow cytometric analysis (n = 2 mice/group).

Supplementary Figure 6

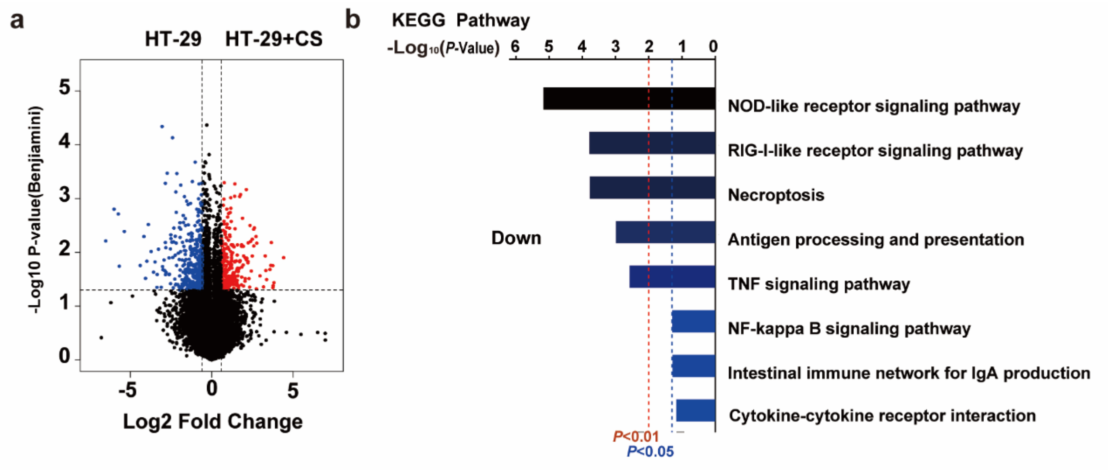

Supplementary Figure 6

a: Volcano plot of the upregulated and downregulated genes from the transcriptomic profiling of HT-29 cells treated with DMSO and HT-29 cells treated with 50 μM CS. (Fold change ≥ 1.5, P<0.05). **b**: KEGG enrichment for downregulated genes in CS-treated HT-29 cells compared with control HT-29 cells (treated with DMSO).

Supplementary Figure 7

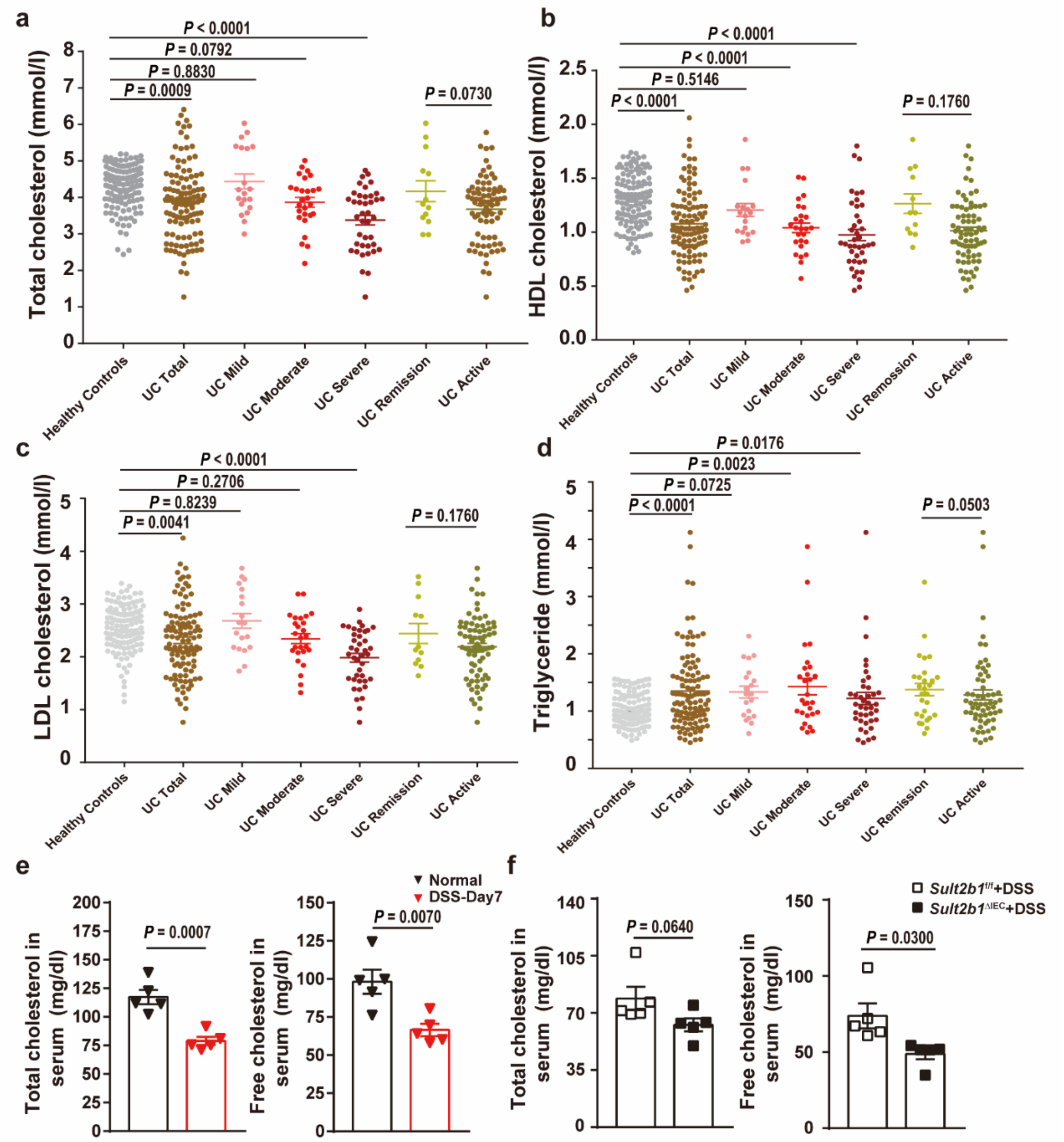

Supplementary Figure 7

a: Serum total cholesterol concentrations of healthy controls (n = 123) and patients with UC (n = 112), patients with mild UC (n = 20), patients with moderate UC (n = 27) and patients with severe UC (n = 40), patients with UC in remission (n = 13) and patients in an active UC state (n = 76). **b:** Serum HDL-cholesterol concentrations of healthy controls (n = 123) and patients with UC (n = 109), patients with mild UC (n = 19), patients with moderate UC (n = 26) and patients with severe UC (n = 40), patients with UC in remission (n = 12) and patients in an active UC state (n = 75). **c:** Serum LDL-cholesterol concentrations of healthy controls (n = 123) and patients with UC (n=109), patients with mild UC (n = 19), patients with moderate UC (n = 26) and patients with severe UC (n = 40), patients with UC in remission (n = 12) and patients in an active UC state (n = 76). **d:** Serum triglyceride (TG) concentrations of healthy controls (n = 123) and patients with UC (n = 111), patients with mild UC (n = 20), patients with moderate UC (n = 28) and patients with severe UC (n = 40), patients with UC in remission (n = 28) and patients in an active UC state (n = 60). **e:** The total cholesterol and free cholesterol concentration in serum from normal C57BL/6J mice, 6-day-2.5% DSS-treated C57BL/6J mice using the Amplex® Red Cholesterol Assay Kit (n = 5 mice/group). **f:** The total cholesterol and free cholesterol concentration in serum from *Sult2b1*$^{f/f}$ and *Sult2b1*$^{\Delta IEC}$ mice with 2.5% DSS challenge using the Amplex® Red Cholesterol Assay Kit (n = 5 mice/group). Statistical significance was determined using one-way ANOVA with Sidak's multiple comparison test (a, b, c, d) or two-tailed Student's t test (e, f). Data are shown as the mean ± SEM. Source data are provided as a Source Data file.

Supplementary Table 1

Patient		1	2	3	4	5	6	7	8	9
Sex/age		F/55	M /53	F /56	F/75	M /65	M /71	M /60	M /46	M /72
Range of lesion		extensive	extensive	extensive	left hemicolitis	extensive	extensive	extensive	extensive	extensive
Clinical typing		chronic recurrent type	chronic recurrent type	chronic recurrent type	chronic recurrent type	chronic recurrent type	chronic recurrent type	chronic recurrent type	chronic recurrent type	chronic recurrent type
Severity		moderate	severe	severe	moderate	severe	severe	severe	moderate	severe
Stage		active	active	active	active	active	active	active	active	active
Treatment history for UC		5-ASA	5-ASA,Prednisolone	5-ASA,Prednisolone Azathioprine	5-ASA	5-ASA	5-ASA,Prednisolone Azathioprine,Thal domide	5-ASA,Predniso one	5-ASA,Prednisolone	5-ASA,Prednisolone
Complications		osteoporosis	respiratory failure, cardiac failure	hypertension	hypoproteinemia	anemia,primary biliary cholangitis	diabetes mellitus type 2, coronary heart disease	osteoporosis	N/A	hypoproteinemia, anemia
Blood count	WBC ($\times 10^9$/L)	5.37	5.41	12.67	7.06	6.89	11.47	7.12	6.39	16.9
	RBC ($\times 10^{12}$/L)	5.08	2.9	3.08	4.11	4.34	4.02	4.49	4.81	3.29
	Hemoglobin (g/L)	148	98	96	113	125	138	122	118	94
	Platelet ($\times 10^9$/μL)	268	40	302	578	324	245	374	496	299
Blood chemistry	ALT (IU/L)	9.85	40.44	25.61	7.8	46	11.9	9.73	16.8	6.23
	AST (IU/L)	13.13	45.36	41.22	14.41	24.24	13.6	16.48	22.52	9.49
	total bililrubin (μmol/L)	11.65	107.26	7.11	7.99	15.16	11.45	7.46	6.95	12.23
	CRP (mg/L)	9.35	55.51	2.83	57.24	112.44	18.93	10	75.47	51.6
	urea (mmol/L)	3.53	3.11	3.63	4.21	8.3	2.89	4.49	4.33	4.19
	creatinine (μmol/L)	51.94	45.88	46.96	55.33	110.93	52.9	63.85	72.29	48.41
	CHO (mmol/L)	3.42	3.37	2.5	4.14	2.67	4.42	/	3.52	2.66
	TG (mmol/L)	4.5	1.42	1.72	1.02	1.25	0.68	/	1.18	1.23
	HDL-C (mmol/L)	1.18	0.52	1.09	1.08	0.69	1.71	/	0.67	0.73
	LDL-C (mmol/L)	1.73	2.45	1.18	2.84	1.73	2.3	/	2.58	1.75

Supplementary Table 1: The demographic and clinical characteristics of the UC patients involved in the immunohistochemical analysis of figure 1b.

Supplementary Table 2

		Ulcerative colitis		Healthy controls	*P*-value
Number of patients	Total	27		19	
	Degree	mild	11		
		moderate	6		
		severe	10		
	range of lension	extensive	11		
		rectum	16		
	Specimen sampling site	rectum	27	19	
Gender	Male		12	7	
	Female		15	12	0.646
Age（mean+SD）			52.1±13.2	54.2±8.9	0.552

Supplementary Table 2: The demographic and clinical characteristics of UC patients and healthy controls involved in the LC-MS analysis of figure 1c and 3g. Categorical data for two groups were analyzed using the Chi-square test.

Supplementary Table 3

Product #D10012G	*gm%*	*Kcal%*
**Protein**	20	20.3
**Carbohydrate**	64	63.9
**Fat**	7	15.8
**Total**		100
**Kcal/gm**	3.9	
**Ingredient**	gm	kcal
**Casein, 30 mesh**	200	800
**l-cystine**	3	12
**Corn starch**	397	1590
**Maltodextrin**	132	528
**Sucrose**	100	400
**Cellulose**	50	0
**Soybean oil**	70	630
**t-butylhydroquinone**	0.014	0
**Mineral mix s10022G**	35	0
**Vitamin mix V10037**	10	40
**Choline bitartrate**	2.5	0
**Total**	1000	4000

Supplementary Table 3: The composition of diet D10012G used in this study.

Supplementary Table 4

Type	*Ingredient*
Control fodder	D10012G
Additional High Cholesterol fodder	D10012G and 1.5% cholesterol
Additional Cholesterol fodder	D10012G and 0.005% cholesterol
Additional CS fodder	D10012G and 0.004% CS

Supplementary Table 4: The information of diets supplemented with cholesterol or cholesterol sulfate.

Supplementary Table 5

Score	*Severity of inflammation*	*Crypt damage*	*Depth of injury*
0	None	None	None
1	Light	Basal one-third damaged,	Mucosal,
2	Moderate	Basal two-thirds damaged,	Mucosal and submucosal
3	Severe	Only surface epithelium intact	Transmural
4		Entire crypt and epithelium lost)	

Supplementary Table 5: Pathological scoring criteria for ulcerative colitis.

Supplementary Table 6

Gene	*Forward (5'-3')*	*Reverse (3'-5')*
h-SULT2B1	CAGTTTGGCTCCTGGTTCG	GAGGCAGCAGCGTGTAGTTG
h-HMGCR	ATAGGAACGGTGGGTGGT	GAGCATCGAGGGTAAACG
h-HMGCS1	GAAGAGTCTGGGAATACA	GAGCGTAAGTTCTTCTGT
h-MVD	CCACAACAGCCGTCATCAGC	CGTGAGTTCCTCCGCTTCC
h-MVK	CCTGCCGAGCCTGGATAT	CGAGGGACTTTGGTGTTGG
h-FDPS	CCCTTTGCTCCTCCCTCA	TGTTTCCTTGGCTCCACC
h-FDFT1	GTGATTGCCGACATTTGC	CTTCTTAACATACCTGCTCC
h-DHCR7	TGTCTGGCTGCCTTATCTT	GGTCCTTCTGGTGGTTGG
h-CYP51A1	CAGTCGCCTGACAACACC	GCCAACCTGGTAAGAGCC
h-SREBF2	CCCATAATATCATTGAGAAACG	TTGTCCACCAGACTGCCTA
h-36B4	CCACGCTGCTGAACAT	CAACATTGCGGACACC
m-SULT2B1	GCGAGACCATCATAAGCG	GTAAATCACCTTAGCCTTGGA
m-36B4	GGGCATCACCACGAAAATCTC	CTGCCGTTGTCAAACACCT
m-IL-1β	GTTCCCATTAGACAACTGC	GATTCTTTCCTTTGAGGC
m-IL-6	TTCTTGGGACTGATGCTG	CTGGCTTTGTCTTTCTTGTT

Supplementary Table 6: Primer sets used for qPCR

Supplementary Table 7

			Ulcerative colitis	*Healthy controls*	*p value*
**Number of patients**	Total		113	123	
	Degree	Mild	20		
		Moderate	27		
		Severe	40		
		Unknown	26		
	Condition	Remission	13		
		Active	76		
		Unknown	24		
**Gender**	Male		70	58	
	Female		43	64	
**Age (mean ± SD)**			53.2 ± 1.35	50.74±1.041	P=0.1049
**BMI (mean ± SD)**			22.26 ± 0.2951	23.76 ± 0.2497	P=0.0001

Supplementary Table 7: The demographic and clinical characteristics of UC patients and healthy controls involved in the retrospective analysis of supplementary figure 6a-d. Categorical data for two groups were analyzed using the Chi-square test.
