# Supplementary material for: Cholesterol sulfate alleviates ulcerative colitis by promoting cholesterol biosynthesis in colonic epithelial cells
Source: Nat Commun. 2022 Jul 30;13:4428. doi: 10.1038/s41467-022-32158-7 (PMC9338998; doi:10.1038/s41467-022-32158-7)
Supplement: Supplementary file 2 — Reporting Summary [file 41467_2022_32158_MOESM2_ESM.pdf]

nature portfolio

Corresponding author(s): Xiaobo Li

Last updated by author(s): Jul 13, 2022

Reporting Summary

Nature Portfolio wishes to improve the reproducibility of the work that we publish. This form provides structure for consistency and transparency in reporting. For further information on Nature Portfolio policies, see our Editorial Policies and the Editorial Policy Checklist.

Statistics

For all statistical analyses, confirm that the following items are present in the figure legend, table legend, main text, or Methods section.

n/a	Confirmed	
☐	☒	The exact sample size (n) for each experimental group/condition, given as a discrete number and unit of measurement
☐	☒	A statement on whether measurements were taken from distinct samples or whether the same sample was measured repeatedly
☐	☒	The statistical test(s) used AND whether they are one- or two-sided<br>*Only common tests should be described solely by name; describe more complex techniques in the Methods section.*
☐	☒	A description of all covariates tested
☐	☒	A description of any assumptions or corrections, such as tests of normality and adjustment for multiple comparisons
☐	☒	A full description of the statistical parameters including central tendency (e.g. means) or other basic estimates (e.g. regression coefficient) AND variation (e.g. standard deviation) or associated estimates of uncertainty (e.g. confidence intervals)
☐	☒	For null hypothesis testing, the test statistic (e.g. F, t, r) with confidence intervals, effect sizes, degrees of freedom and P value noted<br>*Give P values as exact values whenever suitable.*
☒	☐	For Bayesian analysis, information on the choice of priors and Markov chain Monte Carlo settings
☒	☐	For hierarchical and complex designs, identification of the appropriate level for tests and full reporting of outcomes
☐	☒	Estimates of effect sizes (e.g. Cohen's d, Pearson's r), indicating how they were calculated

Our web collection on statistics for biologists contains articles on many of the points above.

Software and code

Policy information about availability of computer code

Data collection	Desorption Electrospray Ionization (DESI) Instrument, XS (Waters, Milford, MA, USA): collecting DESI data.<br>Mass spectrometer Instrument, LTQ Orbitrap XL (Thermo Fisher, San Jose, CA, USA): collecting DESI data.<br>Waters ACOUITY UPLC instrument (Waters, Milford, MA, USA) and Triple quadrupole mass spectrometer API 4000 instrument (Applied Biosystems, SCIEX, USA): collecting LC-MS data.<br>Confocal Laser Scanning Microscope (Leica Microsystems GmbH, DMI6000B)：collecting the immunofluorescence data.<br>FACSCalibur (BD Biosciences): collecting flow cytometry data.<br>Applied Biosystems 7300 Plus Real Time PCR system (ABI): collecting qRT-PCR data.<br>A gel imaging system (Tanon-4200, Shanghai, China): collecting Western-blot data.<br>Hiseq X10 platform (Illumina, San Diego, CA, USA): RNA sequencing
Data analysis	DESI data analysis was performed by Xcalibur 2.2 SP1.48 (Thermo Fisher, San Jose, CA, USA), MATLAB 2021a (Mathworks, Natick, MA, USA), MassImager (Chemmind, Beijing, China).<br>The statistical analyses were performed by GraphPad Prism 8.0 Software (San Diego, CA, USA) and Image J 1.50b software.<br>Primer design was performed by Primer Premier 5.0 software.<br>Flow cytometry data were analysed using FlowJo VX.<br>The mouse genome sequence (mm10) and the human genome sequence (GRCH38) was obtained from the UCSC Genome Browser (http://genome.ucsc.edu/). The annotated gene models were taken from Ensembl (http://www.ensembl.org/). HISAT2 version 2.0.0-beta was used to align the RNA-seq reads from the FASTQ files onto reference genome with default settings. The output files in SAM format were transformed by SAMtools to BAM format. Cuffdiff version 2.2.1 was used to generate FPKM (fragments per kilobase of exon per million reads mapped) data.

For manuscripts utilizing custom algorithms or software that are central to the research but not yet described in published literature, software must be made available to editors and reviewers. We strongly encourage code deposition in a community repository (e.g. GitHub). See the Nature Portfolio guidelines for submitting code & software for further information.

Data

Policy information about availability of data

All manuscripts must include a data availability statement. This statement should provide the following information, where applicable:

- Accession codes, unique identifiers, or web links for publicly available datasets
- A description of any restrictions on data availability
- For clinical datasets or third party data, please ensure that the statement adheres to our policy

The transcriptional expressions of mouse Npc1l1 and human SULT2B1, HMGCS1, MVK, MVD, FDPS, FDFT1, SQLE, LSS, CYP51A1, MSMO1, NSDHL, SC5D, and DHCR7 were analyzed from the published GEO datasets (http://www.ncbi.nlm.nih.gov/geo/). The gene expression profiles of the mouse jejunum, ileum, and colon were obtained from GEO (GSE 143342). The RNA-seq data from patients with clinical UC were from GEO (GSE 111889). Raw data of RNA sequencing generated in this study were deposited at SRA database of NCBI with the accession number PRJNA644070. The gene ontology (GO) and Kyoto Encyclopedia of Genes and Genomes (KEGG) database used in the study are available at http://geneontology.org/ and https://www.genome.jp/kegg/. Gene set enrichment analysis (GSEA) was performed using the GSEA software (http://software.broadinstitute.org/gsea/index.jsp). If needed, contact X.L. for original data described in the paper. Contact X.L. for requesting Sult2b1f/f and Sult2b1ΔIEC mouse strain, and all other plasmids or reagents described in this article. Source data are provided with this paper.

Field-specific reporting

Please select the one below that is the best fit for your research. If you are not sure, read the appropriate sections before making your selection.

☒ Life sciences ☐ Behavioural & social sciences ☐ Ecological, evolutionary & environmental sciences

For a reference copy of the document with all sections, see nature.com/documents/nr-reporting-summary-flat.pdf

Life sciences study design

All studies must disclose on these points even when the disclosure is negative.

Sample size	Sample size estimates has been performed on previous experience to obtain statistical significance and reproducibility. For in vivo studies, the sample size was determined to be enough to obtain the statistical difference between groups, and genotype. Animals were randomly assigned to treatment groups. All sample sizes are listed in the corresponding figure legends or on the figures. All experiments were repeated at least three times.
Data exclusions	When we compared the normalized FPKM of genes of interest in the rectum among non-IBD (n=23) and UC groups(n=26) from published RNA-seq data (GSE 111889), we found the normalized FPKM of SULT2B1 in non-IBD sample "PSM6XBZW" is 0.02416, which is 19.02-fold of the average value of other 22 non-IBD samples (average: 0.00127, SD: 0.00054). Then, when we compared gene expressions in figure 1a and figure 3f, we excluded this outlier (PSM6XBZW) from the non-IBD group in this study. Therefore, 22 non-IBD and 26 UC samples were included for analysis in this study.
Replication	All experiments were conducted at least three times independently, and similar results were adopted for further analysis to guarantee reproducibility.
Randomization	Samples were randomly allocated into the study.
Blinding	This study included a lot of complicated experimental design, the feasibility of blinding was poor, thus blinding was not efficiently applied. The investigators were blinded to group allocation during data collection. Data analysis were performed by different investigators and analysis to avoid conscious and unconscious bias.

Reporting for specific materials, systems and methods

We require information from authors about some types of materials, experimental systems and methods used in many studies. Here, indicate whether each material, system or method listed is relevant to your study. If you are not sure if a list item applies to your research, read the appropriate section before selecting a response.

Materials & experimental systems

n/a	Involved in the study	
	x	Antibodies
	x	Eukaryotic cell lines
x		Palaeontology and archaeology
	x	Animals and other organisms
	x	Human research participants
x		Clinical data
x		Dual use research of concern

Methods

n/a	Involved in the study	
x		ChIP-seq
	x	Flow cytometry
x		MRI-based neuroimaging

Antibodies

Antibodies used

anti-human SULT2B1 (R&D Systems, Cat. #AF6174, 1:1000)
anti-mouse SULT2B1 (Santa Cruz Biotechnology, Cat. #sc-166423, 1:1000)
anti-human SREBP2 (ABclonal, Cat. #A13049, 1:1000)
HA-tag rabbit mAb (Cell Signaling Technology, #3724),
anti-human SLC10A6 (Invitrogen, Cat. #PA5-53468, 1:200)
anti-β-actin (Proteintech, Cat. #66009-1, 1:2000)
anti-HSP90 (Proteintech, Cat. #60318-1-Ig, 1:2000)
anti-human NPC2 (Proteintech, Cat. #19888-1-AP, 1 ： 1000)
Monoclonal ANTI-FLAG® M2 antibody (Sigma-Aldrich, Cat. # F1804, 1:2000)
peroxidase-conjugated goat anti-rabbit secondary antibody (Yeasen, Cat. #33101ES60, 1:2500)
peroxidase-conjugated goat anti-mouse secondary antibody (Yeasen, Cat. #33201ES60, 1:2500)
peroxidase-conjugated rabbit anti-sheep secondary antibody (Abcam, Cat. #ab6747, 1:2500).
CD45 (Invitrogen, Cat. #11-0451-82, 1:100)
CD19 (Invitrogen, Cat. #17-0193-80,1:100)
Ly-6G/6C(Invitrogen, Cat. #25-5931-81, 1:100)
CD11b (Invitrogen, Cat. #17-0112-81, 1:100)
CD3e (Invitrogen, Cat. #17-0031-81, 1:100)
CD4 (Invitrogen, Cat. #12-0041-81, 1:100)
CD8a (Invitrogen, Cat. #25-0081-81, 1:100)

Validation

Validation of the use of anti-human SULT2B1 antibody for human in Western blot and immunohistochemistry has been provided by the manufacturer’s website.

Validation of the use of anti-mouse SULT2B1 antibody for mouse in Western blot has been provided by the manufacturer’s website.

Validation of the use of SREBP2 antibody for human in immunostaining has been provided by the manufacturer’s website.

Validation of the use of HA antibody for human in Western blot has been provided by the manufacturer’s website.

Validation of the use of SLC10A6 antibody for human in immunostaining have been provided by the manufacturer’s website.

Validation of the use of β-actin and HSP90 for human and mouse in Western blot has been provided by the manufacturer’s website.

Validation of the use of NPC2 for human in Western blot has been provided by the manufacturer’s website.

Validation of the use of FLAG antibody for human in Western blot has been provided by the manufacturer’s website.

Validation of the use of goat anti-rabbit secondary antibody, goat anti-mouse secondary antibody and peroxidase-conjugated rabbit anti-sheep secondary antibody in Western-blot has been provided by the manufacturers’ website.

Validation of the use of antibodies (including CD45, Ly-6G/Ly-6C, CD11b, CD19, CD3e, CD4, and CD8a) for mouse in flow cytometry has been provided by the manufacturer’s website.

Eukaryotic cell lines

Policy information about cell lines

Cell line source(s)

293T cell line, human colon carcinoma cell lines HT-29, LOVO, SW480, HCT116, and SW1116 were from National Collection of Authenticated Cell Cultures (Shanghai, China, Cat#SCSP-502, SCSP-5032, #SCSP-514, #SCSP-5033, #TCHu99, #TCHu174,

	respectively).
Authentication	STR testing
Mycoplasma contamination	All the cell lines were test negative for the mycoplasma contamination.
Commonly misidentified lines (See ICLAC register)	No commonly misidentified cell lines were used in the study.

Animals and other organisms

Policy information about studies involving animals; ARRIVE guidelines recommended for reporting animal research

Laboratory animals	Male C57BL /6J mice, aged 8-12 weeks; Sult2b1f/f mice and Villin-Cre (Sult2b1ΔIEC) mice(male or female, 8-12 weeks, Age- and sex-matched in the study); Male C57BL/6 interleukin-10 deficient (IL-10-/-) mice, aged 8-12 weeks.
Wild animals	The study did not involve wild animals.
Field-collected samples	The study did not involve field-collected samples
Ethics oversight	All animal studies were performed according to protocols approved by the Animal Ethics Committee of the Fudan University School of Basic Medical Sciences.

Note that full information on the approval of the study protocol must also be provided in the manuscript.

Human research participants

Policy information about studies involving human research participants

Population characteristics	The demographic and clinical characteristics of the UC patients involved in the immunohistochemical analysis of figure 1b were provided in Supplementary Table 1.<br>The demographic and clinical characteristics of UC patients and healthy controls involved in the LC-MS analysis of figure 1c and 3g were provided in Supplementary Table 2.<br>Supplementary Table 7: The demographic and clinical characteristics of UC patients and healthy controls involved in the retrospective analysis of supplementary figure 6a-d were provided in Supplementary Table 7.
Recruitment	Patients in this study were recruited from Shanxi Provincial People's Hospital, Taiyuan, China. All patients information and samples were collected based on clinical requirement for diagnosis. There were no self-selection bias or other biases.
Ethics oversight	All patients gave informed consents for collection of tissue collection. All procedures were performed in accordance with institutional guidelines and were approved by Shanxi Provincial People's Hospital Research Ethics Committee with the reference number 2019-70 for this study. The study was conducted in accordance with the criteria set by the Declaration of Helsinki.

Note that full information on the approval of the study protocol must also be provided in the manuscript.

Flow Cytometry

Plots

Confirm that:

- [x] The axis labels state the marker and fluorochrome used (e.g. CD4-FITC).
- [x] The axis scales are clearly visible. Include numbers along axes only for bottom left plot of group (a 'group' is an analysis of identical markers).
- [x] All plots are contour plots with outliers or pseudocolor plots.
- [x] A numerical value for number of cells or percentage (with statistics) is provided.

Methodology

Sample preparation	Split the colon after removing the Peyer's patch in it. Cut the colon into 0.5 cm fragment and put them into CMF solution (HEPES (2.6 g/L), NaHCO3 (2.1 g/L), EDTA (0.29 g/L), DTT (0.15 g/L), FCS (4 ml), HBSS (to 1000 ml). 37 oC, 220rpm, 1h. Discard the supernate and put the colon fragment into Digestion solution (10% 1640 (1ml), collagenase (0.5 mg/ml), Dispase (20 uL), DNase (2 uL)). 37 oC, 220 rpm, 45 mins. Filter into new tube and washed by PBS for 2 times. Resuspend the cell suspension into 5 ml 40% percoll. Add 5 ml 70% percoll into a new 15 ml tube and add 5 ml cell suspension that was resuspended in 40% percoll. Density gradient centrifugation, 220 rpm, 30 mins. Get the lamina propria lymphocytes of the colon between the 70% percoll and 40% percoll. Lamina propria lymphocytes of the colon were incubated with CD45 (Invitrogen, Cat. #11-0451-82), Ly-6G/Ly-6C (Invitrogen, Cat. #25-5931-81), CD11b (Invitrogen, Cat. #17-0112-81), CD11c (Invitrogen, Cat. #12-0114-81), CD3e (Invitrogen, Cat. #17-0031-81), CD45R (Invitrogen, Cat. #12-0452-81), CD4 (Invitrogen, Cat. #12-0041-81), and CD8a (Invitrogen, Cat. #25-0081-81) antibodies, respectively.
Instrument	FACSCalibur (BD Biosciences)

Software	FlowJo VX
Cell population abundance	A total of 20,000 gated events were counted for each marker in three independent experiments.
Gating strategy	Double Positive cells were identified by comparing with the blank and single straining samples.

☒ Tick this box to confirm that a figure exemplifying the gating strategy is provided in the Supplementary Information.
